# Rhizosphere heterogeneity shapes abundance and activity of sulfur-oxidizing bacteria in vegetated salt marsh sediments

**DOI:** 10.3389/fmicb.2014.00309

**Published:** 2014-06-24

**Authors:** François Thomas, Anne E. Giblin, Zoe G. Cardon, Stefan M. Sievert

**Affiliations:** ^1^Watson Laboratory, Biology Department, Woods Hole Oceanographic Institution, Woods HoleMA, USA; ^2^Marine Biological Laboratory, The Ecosystems Center, Woods HoleMA, USA

**Keywords:** *rdsr*AB, rhizosphere, salt marsh, *sox*B, sulfur oxidation, *Spartina alterniflora*

## Abstract

Salt marshes are highly productive ecosystems hosting an intense sulfur (S) cycle, yet little is known about S-oxidizing microorganisms in these ecosystems. Here, we studied the diversity and transcriptional activity of S-oxidizers in salt marsh sediments colonized by the plant *Spartina alterniflora*, and assessed variations with sediment depth and small-scale compartments within the rhizosphere. We combined next-generation amplicon sequencing of 16S rDNA and rRNA libraries with phylogenetic analyses of marker genes for two S-oxidation pathways (*sox*B and *rdsr*AB). Gene and transcript numbers of *sox*B and *rdsr*AB phylotypes were quantified simultaneously, using newly designed (RT)-qPCR assays. We identified a diverse assemblage of S-oxidizers, with *Chromatiales* and *Thiotrichales* being dominant. The detection of transcripts from S-oxidizers was mostly confined to the upper 5 cm sediments, following the expected distribution of root biomass. A common pool of species dominated by *Gammaproteobacteria* transcribed S-oxidation genes across roots, rhizosphere, and surrounding sediment compartments, with *rdsr*AB transcripts prevailing over *sox*B. However, the root environment fine-tuned the abundance and transcriptional activity of the S-oxidizing community. In particular, the global transcription of *sox*B was higher on the roots compared to mix and rhizosphere samples. Furthermore, the contribution of *Epsilonproteobacteria*-related S-oxidizers tended to increase on *Spartina* roots compared to surrounding sediments. These data shed light on the under-studied oxidative part of the sulfur cycle in salt marsh sediments and indicate small-scale heterogeneities are important factors shaping abundance and potential activity of S-oxidizers in the rhizosphere.

## Introduction

Salt marshes are highly productive coastal ecosystems found in intertidal areas and vegetated by salt tolerant non-woody plants. Along the Atlantic coast of the United States, the cord grass *Spartina alterniflora* is typically the dominant plant in areas that are submerged for part of each tidal cycle (Niering and Warren, 1980). Rates of net primary production by *S. alterniflora* are extremely high, ranging from 460 to 3200 g cm^−2^.year^−1^, much of which occurs belowground (Schubauer and Hopkinson, 1984; Giblin and Wieder, 1992). The high concentration of organic matter produced either by the decay of plant tissues or leakage of dissolved compounds from the root system, fuels aerobic respiration and, together with frequent waterlogging, leads to oxygen depletion in the sediments. Thus, abundant residual organic matter is available for anaerobic respiration through sulfate reduction. Consequently, rates of sulfate reduction in salt marshes are among the highest reported in the marine environment (Howarth and Hobbie, 1982; Hines et al., 1989; Laverman et al., 2012). The sulfide produced by sulfate reduction still contains much of the photochemical energy that was initially captured by plants into carbon-carbon bonds, making it potentially available to organisms collectively known as sulfur-oxidizers (S-oxidizers), completing the sulfur cycle (Howarth and Teal, 1980).

Sulfate-reducing prokaryotes have previously been extensively studied in salt marsh systems and were found to be generally dominated by *Deltaproteobacteria*, in particular members of the family *Desulfobacteraceae* and *Desulfobulbaceae* (Devereux et al., 1996; Klepac-Ceraj et al., 2004; Bahr et al., 2005). In contrast very little is known about the organisms carrying out S-oxidation in salt marshes. To date, S-oxidizers are known dominantly from environments such as hydrothermal vents, brines and non-vegetated coastal sediments (Robertson and Kuenen, 2006; Sievert et al., 2007; Ghosh and Dam, 2009). Only a few S-oxidizers have been cultivated from salt marshes, such as *Thiovulum* (Wirsen and Jannasch, 1978), *Beggiatoa* (Nelson et al., 1982) and *Candidatus* Arcobacter sulfidicus (Wirsen et al., 2002). In neutrophilic S-oxidizers, two distinct sulfur oxidation pathways involving different enzymes exist. Through the multi-enzyme SOX complex composed of SoxYZ, SoxXA, SoxB, and SoxCD, reduced sulfur compounds are completely oxidized to sulfate (Kelly et al., 1997; Friedrich et al., 2001). Alternatively, bacteria lacking the SoxCD component use the branched thiosulfate oxidation pathway, whereby sulfur accumulates as an intermediate and is sequentially oxidized to sulfite and sulfate by a reverse acting dissimilatory sulfite reductase (rDSR), an APS reductase and an ATP sulfurylase (Pott and Dahl, 1998; Kappler and Dahl, 2001). Genes involved in these two pathways have been used as functional markers to study the diversity and distribution of S-oxidizers in the environment, namely *sox*B (Petri et al., 2001; Meyer et al., 2007; Akerman et al., 2013), *rdsr*AB (Loy et al., 2009; Lenk et al., 2011) and *apr*A (Meyer and Kuever, 2007).

Many S-oxidizers are autotrophs or facultative autotrophs. They can couple S-oxidation to carbon fixation, therefore releasing the energy trapped in sulfide or other reduced sulfur compounds and producing labile organic matter that can fuel higher trophic levels. The lower concentrations of sulfide often reported from various salt marshes compared to subtidal sediments (Giblin and Howarth, 1984) suggest high rates of sulfide turnover, which may be enabled by a tight interaction between the microorganisms and the plants. Oxygen can diffuse out of plant roots into the sediment (Holmer et al., 2002), where S-oxidizers colonizing the rhizosphere may use it as a terminal electron acceptor. This interaction could benefit the plant by removing hydrogen sulfide, which is known to inhibit plant growth (Joshi and Hollis, 1977; Bradley and Morris, 1990; Pezeshki and Delaune, 1996). Alternatively, S-oxidizers can use nitrate as the electron acceptor and produce either N_2_ via denitrification or NH^+^_4_ via dissimilatory nitrate reduction to ammonium (Timmer-Ten Hoor, 1975; Brunet and Garcia-Gil, 1996; Otte et al., 1999; Sayama et al., 2005; Burgin and Hamilton, 2007) potentially affecting the fate of nitrate in eutrophic systems. Thus, the salt marsh rhizosphere is a dynamic, metabolically active environment featuring a variety of contrasted small-scale compartments where microorganisms involved in reductive and oxidative portions of S-cycling can co-exist and couple S-cycling with nitrogen and carbon cycling.

The present study investigates bacterial communities of S-oxidizers in sediments colonized by the dominant plant *S. alterniflora* in a New England salt marsh. We studied how small-scale heterogeneities across the vegetated sediment affect the diversity, distribution, and transcriptional activity of S-oxidizers. Specifically, we hypothesized that the rhizosphere or rhizoplane would represent an area of enhanced activity compared to bulk sediment due to a higher availability of electron acceptors to support the oxidation of reduced sulfur compounds and the possible interactions between S-oxidizers and sulfate-reducers, which have been shown to be stimulated by the release of dissolved organic matter from the roots. We further hypothesized that S-oxidizers known to be adapted to higher oxygen concentrations, like *Gammaproteobacteria*, would be found predominantly closer to the roots, while S-oxidizers known to be adapted to lower oxygen concentrations, like *Epsilonproteobacteria*, would be found further away. Samples were taken at two sites selected for their different flooding and salinity regimes. At each site, we collected rhizosphere and root samples separately in addition to bulk sediment samples. To characterize the S-oxidizers, we combined amplicon sequencing of 16S rDNA and rRNA libraries with phylogenetic analyses of marker genes for two S-oxidation pathways (*sox*B and *rdsr*AB). The abundance and transcriptional activity of the S-oxidizers identified were then quantified using newly designed (RT)-qPCR assays for the *sox*B and *rdsr*AB genes.

## Materials and methods

### Site descriptions

Samples were collected from two locations vegetated with *S. alterniflora*, selected for contrasting flooding and salinity regimes. The sites were 1.5 km apart along the Rowley River (Massachusetts, USA) at Plum Island Ecosystems Long-Term Ecological Research (PIE-LTER). Samples from Site 1 (N 42° 43′ 32.04”, W 70° 51′ 19.91”) were taken at the creekbank, characterized by more frequent and longer tidal flushing than the second site. Samples from Site 2 (N 42° 43′ 56.92”, W 70° 50′ 26.18”), which was further downstream, were collected on the marsh platform 9 m from the creekbank. Salinities at Site 2 during a typical summer range from 28–32 psu and show little variation over the tidal cycle. Salinities vary more at the Site 1 and range from 23 to 31 psu.

### Sample collection

For the investigation of bacterial diversity and transcriptional activity with depth in sediment, one core (polycarbonate core liner, 9 cm diameter, 30 cm length) was retrieved from Site 2 during July 2012, and subsampled at depth intervals of 2 cm (from 0 to 20 cm) within 2 h. Two grams of sediment/root mixture from each depth (hereafter referred to as “mix” samples) were transferred to a sterile tube containing 6 ml of LifeGuard (MO-BIO, Carlsbad, CA). For the exploration of diversity and transcriptional activity on and around roots, we collected three cores at Site 1 and three at Site 2 in October of 2012. Cores were taken 50 cm apart. Within 2 h, “mix,” “rhizosphere” and “roots” subsamples were taken from each core, at 5 cm depth. “Mix” samples were similar to those taken in July. Rhizosphere samples were gathered by separating roots from the remaining sediment with tweezers and gently shaking them in LifeGuard, releasing adhered “rhizosphere” sediment (0.2–1 g). Shaken roots were transferred to another tube containing LifeGuard, resulting in the “roots” sample (0.2–0.5 g). All samples were frozen on dry ice and stored at −20°C. To minimize bias, the same manipulator performed all sampling and root-sediment separation procedures.

### Nucleic acid extraction and cDNA synthesis

RNA and DNA were extracted simultaneously using the RNA PowerSoil Total RNA Isolation and DNA Elution Accessory kits (MO-BIO) following the manufacturer's instructions for “mix” and “rhizosphere” samples. For consistency, the same kits were used to extract RNA and DNA from “roots” samples, placing the intact roots in the bead beating tubes. DNA was further purified using the PowerClean DNA Clean-Up kit (MO-BIO). RNA was digested for 30 min at 37°C with 2 units of TURBO-DNase (Ambion, Austin, TX) and purified using the RNeasy MinElute Cleanup kit (Qiagen, Valencia, CA). Reverse-transcription reactions were performed on 800 ng (mix and rhizosphere samples) or 400 ng (roots samples) of total RNA using the DyNAmo cDNA Synthesis kit (Thermo Scientific). No-RT control reactions including all components except for the reverse transcriptase were prepared for each sample. cDNA synthesis was checked by PCR using bacterial 16S rRNA gene primers S-D-Bact-0341-a-S-17 and S-D-Bact-0515-a-A-19 (Klindworth et al., 2012). No PCR products were detected for similar reactions with no-RT controls, confirming the absence of gDNA contamination.

### Illumina tag sequencing and analysis

Amplicon libraries were produced from DNA and cDNA fractions for all eighteen October samples, and five of the ten July samples (distributed from surface to depth). The V6 region of 16S rRNA genes was amplified using previously reported primers designed for bacteria (Huber et al., 2007). Triplicate PCR reactions were conducted on 25 ng of template in a final volume of 25 μl, containing 2 mM MgSO_4_, 0.2 mM dNTPs, 0.4 μM of each primer, 1X Hi-Fidelity buffer and 2 U of Platinum Taq Hi-Fidelity Polymerase (Life Technologies, Carlsbad, CA). Reactions were denatured for 3 min at 94°C, followed by 25 cycles of 30 s at 94°C, 45 s at 60°C and 1 min at 72°C, and a final extension of 2 min at 72°C. Triplicate reactions were pooled and purified using a Qiaquick PCR 96-well PCR clean-up plate or MinElute kit (Qiagen, Valencia CA). A second run of PCR (5 cycles) was performed on 8 μl of purified products with fusion primers including indices and barcodes compatible with the Illumina HiSeq1000 platform (Eren et al., 2013).

After quantification by Picogreen assay (Life Technologies), 25 ng of products were pooled and size selected (210–240 bp) on a 2% agarose PippinPrep cassette. The libraries were cleaned using MinElute kits and sequenced at MBL (Woods Hole, MA) in a single paired-end lane of Illumina. Completely overlapping paired-end reads were kept for further analysis (Eren et al., 2013). Quality-filtered reads are available through VAMPS (http://vamps.mbl.edu) (project FRT_PIE1_Bv6). Sequences were clustered at 97% similarity using usearch (Edgar, 2010). Taxonomy was assigned to each Operational Taxonomic Unit (OTU) using GAST (Huse et al., 2008) with a version of the Greengenes 13_5 database (McDonald et al., 2012) trimmed to the V6 region. OTUs were analyzed with QIIME v1.7 (Caporaso et al., 2010) and the R package phyloseq (McMurdie and Holmes, 2013). Data sets were rarefied to the lowest number of sequences per sample (142,916 reads). Bray-Curtis distance was used for Principal Coordinates Analysis (PCoA) on a relative abundance matrix comprising all OTUs belonging to the orders listed in Table 1. LEfSe (Segata et al., 2011) was used on the same matrix to determine indicator bacterial groups with a logarithmic linear discriminant analysis (LDA) score threshold of 2.

**Table 1:** **Number of reads, OTUs, diversity indices and relative abundance ranges of selected bacterial taxa with known sulfur-oxidizing capabilities**.

	**Number of reads**	**Number of OTUs**	**Chao1**	**Shannon**	**Relative abundance (%)**	
					**rDNA**	**rRNA**
**Full dataset**	**14,173,928**	**48575**	**–**	**–**	**–**	**–**
**Rarefied dataset**	**6,574,136**	**46280**	**48181**	**7.94**	**–**	**–**
***Chlorobi***	**175,727**	**510**	**525**	**3.59**	**1.90–4.09**	**1.36–3.02**
*Chlorobiales*	2044	10	10	0.70	0.01–0.06	0.01**–**0.08
***Alphaproteobacteria***	**334,184**	**2710**	**2842**	**5.71**	**3.63–8.14**	**2.06–6.84**
*Rhizobiales*	74,355	627	675	4.59	0.80–1.83	0.51–1.27
*Rhodobacterales*	111,219	671	691	4.42	1.17–2.32	0.65 – 2.27
*Rhodospirillales*	69,150	567	587	4.03	0.60–1.85	0.45–1.53
***Betaproteobacteria***	**50,066**	**393**	**408**	**3.90**	**0.54–1.13**	**0.33–1.35**
*Hydrogenophilales*	1459	5	5	0.87	0.01–0.05	0.01–0.07
*Burkholderiales*	18,659	139	144	2.58	0.11–0.82	0.12–0.88
***Epsilonproteobacteria***	**24,991**	**264**	**278**	**4.07**	**0.17–0.77**	**0.25–0.48**
*Campylobacterales*	24,988	263	277	4.06	0.16–0.77	0.25–0.48
***Gammaproteobacteria***	**1,356,406**	**5611**	**5859**	**5.18**	**16.11–25.49**	**15.67–29.04**
*Chromatiales*	576,136	1633	1681	3.98	6.35–10.44	5.54–14.79
*Thiotrichales*	174,105	612	637	3.29	1.79–4.75	1.67–3.28
*Thiohalorhabdus*-related	101,270	111	117	1.20	1.00–2.55	1.29–3.92

### *Sox*B and *rdsr*AB clone libraries

Amplification of *sox*B and *rdsr*AB genes were performed on 1 ng of DNA from “mix” samples collected in July 2012. *sox*B fragments (~1020 bp) were amplified with the primers soxB432F and soxB1446B, using a previously described two-step PCR (Petri et al., 2001). Reactions contained 2.5 mM MgCl_2_, 0.2 mM of each dNTP, 1 μM of each primer, 0.3 mM BSA, 10% DMSO and 0.5 U of Taq DNA polymerase (Promega). The primers sox527F and sox1198R were used to specifically amplify *sox*B fragments from *Epsilonproteobacteria* (~672 bp) as described previously (Akerman et al., 2013). Reactions contained 2.5 mM MgCl_2_, 0.2 mM of each dNTP, 0.6 μM of each primer and 0.5 U of Taq DNA polymerase. *rdsr*AB gene fragments (~2 kb) were amplified using the primers rDSRA240F and rDSRB808R as described previously (Lenk et al., 2011). PCR reactions contained 1.5 mM MgCl_2_, 0.25 mM of each dNTP, 2 μM of each primer and 0.5 U of Taq DNA polymerase. For each depth and primer set, triplicate reactions (15 μl) were pooled and analyzed by 1% agarose gel electrophoresis. Bands of the expected size were excised and purified using the Qiaquick Gel Extraction Kit. Products from all depths were pooled, cloned using the TOPO-TA cloning kit for sequencing (Invitrogen, Carlsbad, CA) and sequenced by Beckman Coulter Genomics. An internal primer DSR874F was used for sequencing the internal part of the 2 kb *rdsr*AB fragment (Loy et al., 2009). Sequences were checked with Sequencher 5.0.1 (Gene Codes Corporation, Ann Arbor, MI), translated into amino acid sequences and clustered into 90% identity OTUs using the program CD-HIT (Huang et al., 2010). Alignments were generated with MAFFT (Katoh, 2002) and refined manually in MEGA5 (Tamura et al., 2011). Final alignments comprised 206 and 460 amino acid positions for soxB and rdsrAB, respectively. Phylogenetic trees were constructed with the maximum likelihood method in MEGA5. The sequence data have been submitted to the GenBank database under accession No. KF910786-KF910914 (*sox*B) and KF910915-KF910961 (*rdsr*AB).

### Quantitative PCR (qPCR)

The AlleleID 7.75 software (PREMIER Biosoft) was used on alignments of *soxB* and *rdsr*AB sequences to design a suite of primers targeting divergent phylotypes (Table Supp1). Primers were checked against the nr/nt database using Primer-BLAST on NCBI server (Ye et al., 2012). For each target phylotype, DNA standards were prepared by linearizing plasmids from one representative clone. qPCR reactions were performed on a Mx3005P thermocycler (Stratagene) using the Maxima SYBR Green qPCR Master Mix containing 0.01 μM ROX (Thermo Scientific). For each primer set, qPCR conditions were optimized on serial dilution of the respective standard clone (10–10^5^ copies), to ensure satisfying specificity and efficiency above 80%. Reactions were denatured 10 min at 95°C, followed by 40 cycles of 15 s at 95°C, 30 s at the specific annealing temperature and 30 s at 72°C. Dissociation curves were obtained by heating up the reactions from 65 to 95°C. PCR efficiency was determined using the standard curve by the formula *E* = 100^*^(10^(−1/slope)^-1). To check the specificity of each primer set, qPCR reactions were run using either 1 ng of environmental DNA, a mixture of 10^4^ copies of all standard clones or the same mixture without the target standard clone as template. No amplification was detected in the latter case. For the former two cases, a single band of the expected size was observed on 2% (wt/vol) agarose gel. Optimized primer concentrations and cycling conditions are given in Table Supp2. Validated qPCR conditions were used on environmental samples, using 1 ng of DNA or 3 ng cDNA (eq.RNA) as template. For each phylotype, reactions were technically duplicated and fresh standard curves were run on the same plate as environmental samples, to determine assay performances (Table Supp2). A single lot of cDNAs was used to minimize the variability due to reverse-transcription. To ensure no contribution of the background signal to gene quantification, C_T_ cut-off thresholds were set 3.3 cycles lower than that for the no-template control, if detected (Smith et al., 2006). Correlation analysis, ANOVA and *post-hoc* Tukey tests were performed on log_10_(x + 1)-transformed values in R v3.0.1 (http://www.R-project.org/).

## Results

### Taxonomic composition

To identify the major S-oxidizers in salt marshes, we used both phylogenetic analyses of 16S rRNA and genes involved in S-oxidation (*sox*B and *rdsr*AB).

Over 14 million reads were retrieved from bacterial 16S rDNA and rRNA gene amplicon libraries prepared from 23 samples, including all samples from October and 5 selected samples distributed along the depth profile of the July core (Table Supp3). A total of 48,575 OTUs were obtained at the 3% clustering level. We focused on orders in the phyla *Chlorobi* and *Proteobacteria* that are currently known to contain S-oxidizers (Table 1). The most prevalent were *Chromatiales* and *Thiotrichales* within the *Gammaproteobacteria*, and *Rhodobacterales* within the *Alphaproteobacteria.* Twenty-nine of the 100 most abundant OTUs belonged to one of these orders (Table 2). Though not the focus of our work, we identified *Desulfobacteraceae* and *Desulfobulbaceae* as the dominant sulfate-reducing bacteria (unpubl. data), similar to previous studies in salt marshes, including at PIE (Klepac-Ceraj et al., 2004; Bahr et al., 2005).

**Table 2:** **Potential S-oxidizer-affiliated OTUs within the 100 most abundant OTUs of the entire dataset**.

**Rank**	**OTU #**	**Reads number**	**Greengenes taxonomy**	**GAST hit**	
				**Genbank ID**	**Environment**
3	47537	75793	*Chromatiales*; *Chromatiaceae*	AM882561	Coastal sediment from oil polluted water
7	26	45073	*Thiohalorhabdus*-related	AM259913	Sponge mesohyl, Adriatic Sea
8	38	43425	*Thiohalorhabdus*-related	AB694476	Deep-sea sediment at a depth 7111 m
10	18	42564	*Thiotrichales*; *Thiotrichaceae*	AM882526	Coastal sediment from oil polluted water
15	46316	32517	*Chromatiales*; *Ectothiorhodospiraceae*	JX240444	Coastal soil of Gulf of Khambhat
16	46	32367	*Chromatiales*	JN825489	Microbialites from Alchichica alkaline lake maintained in aquarium
19	29	30098	*Chromatiales*; *Chromatiaceae*	JF344607	Hydrocarbon polluted marine sediments
22	14	28188	*Chromatiales*	AF170422	Shallow water hydrothermal vent
26	45	24393	*Chromatiales*	HQ190997	Janssand intertidal sediment; German Wadden Sea
27	28	23825	*Chromatiales*	FN553596	Logatchev hydrothermal vent
29	41591	19911	*Chromatiales*	U77479	Bacterial endosymbiont from Lamellibrachia sp.,Gulf of Mexico seep
32	15	18751	*Chromatiales*	FJ497626	Vailulu'u seamount
37	23	17739	*Chromatiales*; *Chromatiaceae*; *Marichromatium*	FN995224	Seashore sediment
38	64	17322	*Chromatiales*	DQ351776	Marine sediments
41	39	16299	*Thiotrichales*; *Thiotrichaceae*	JF344477	Hydrocarbon polluted marine sediments
52	37	13622	*Chromatiales*; *Ectothiorhodospiraceae*	GQ259300	Surface sediment from Arctic fjord
57	42	12407	*Chromatiales*	JF344456	Hydrocarbon polluted marine sediments
66	76	11515	*Rhodobacterales*; *Rhodobacteraceae*	DQ015815	Lake Bonney water. Antarctica
70	52	11125	*Thiotrichales*; *Piscirickettsiaceae*	JQ580025	Sediments from Figueiras beach
72	53	10863	*Thiotrichales*; *Piscirickettsiaceae*	AB694467	Deep-sea sediment at a depth 7111m
74	102	10805	*Chromatiales*; *Chromatiaceae*	AM882562	Coastal sediment from oil polluted water
76	85	10555	*Chromatiales*	HQ191056	Janssand intertidal sediment; German Wadden Sea
78	78	10316	*Chromatiales*	EF999348	Pearl River estuary sediments at 6 cm depth
81	87	10085	*Chromatiales*	EU488541	Gill symbiont from lucinid clam in sea grass beds
87	68	9431	*Rhodospirillales*; *Rhodospirillaceae*	JX240999	Coastal soil of Gulf of Khambhat
93	79	8794	*Rhodospirillales*; *Rhodospirillaceae*	EU834757	Lab scale EBPR-activated sludge
95	73	8393	*Thiotrichales*; *Piscirickettsiaceae*	JN166335	Hawaii Ocean Time series, depth = 350 m
98	104	8154	*Rhodobacterales*; *Rhodobacteraceae*	JN435530	Guerrero negro hypersaline mat
100	28653	8142	*Chromatiales*; *Chromatiaceae*	FJ437964	Green lake surface sediments at 16.5m water depth

**Figure 1:**
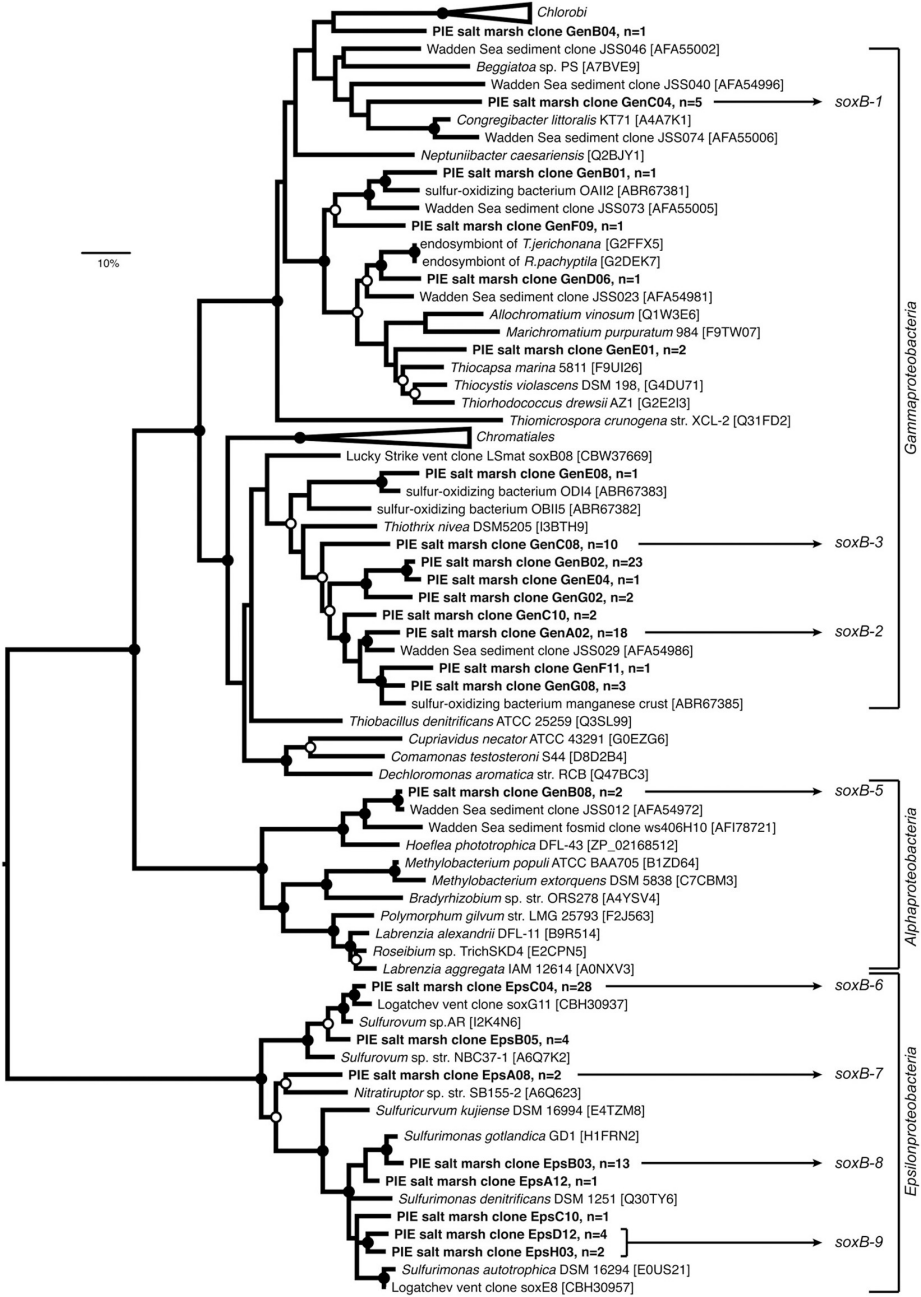
**Maximum-likelihood (ML) phylogenetic reconstruction of SoxB proteins deduced from sequences cloned from Plum Island Estuary salt marsh sediments (in boldface), including publicly available SoxB sequences from reference strains and uncultured bacteria (accession numbers are given)**. The WAG+G substitution model was used (100 re-samplings, *G* = 1.21, *I* = 0.10) based on testing different models in MEGA5. OTUs defined at 90% identity threshold are represented by one selected clone; “*n*” equals number of sequences per OTU. Sequences annoted as “Gen” and “Eps” were retrieved from the clone libraries prepared using the general and *Epsilonproteobacteria*- specific *sox*B primer pairs, respectively. ML bootstrap support (100 resamplings) greater than 50% (open circles) and 70% (black circles) are displayed. The tree was rooted on the *Epsilonproteobacteria* branch. The bar indicates 10% sequence divergence. OTUs used to design *sox*B primer sets targeting selected phylotypes for qPCR are shown (*sox*B 1–9, Table Supp1).

In total 129 *sox*B sequences were retrieved from different sediment layers in July at Site 2, including 74 sequences with the general primers and 55 sequences with the *Epsilonproteobacteria*-specific primers (Figure 1). At 90% amino acid identity, 24 distinct OTUs were identified (9 were singletons). Rarefaction analysis suggested the *sox*B diversity was not completely captured when pooling sequences from both libraries (Figure Supp1).

Phylogenetic reconstruction affiliated the majority of *sox*B sequences with *Gammaproteobacteria* (71 sequences, 14 OTUs). Fifty-five sequences (8 OTUs) were related to *Epsilonproteobacteria* and only 2 sequences (1 OTU) clustered with *Alphaproteobacteria.* One *sox*B singleton was distantly related to *Chlorobi*, and none of the retrieved sequences clustered with known *Betaproteobacteria* S-oxidizers. Although *Thiohalorhabdus*-related sequences were found relatively abundant in the 16S rRNA libraries (Table 1), they were not detected in the *sox*B library (Figure 1). Using the same primers as we did, Tourova et al. (2013) successfully amplified the *sox*B gene from the extreme halophile *Thiohalorhabdus denitrificans*, an unclassified gammaproteobacterial S-oxidizer with no significant relationship to any other genera in this class (Sorokin et al., 2008). Therefore, *Thiohalorhabdus*-related bacteria detected in the 16S rRNA libraries might contain either a divergent *sox*B or no *sox*B at all and may not be involved in sulfur oxidation.

**Figure 2:**
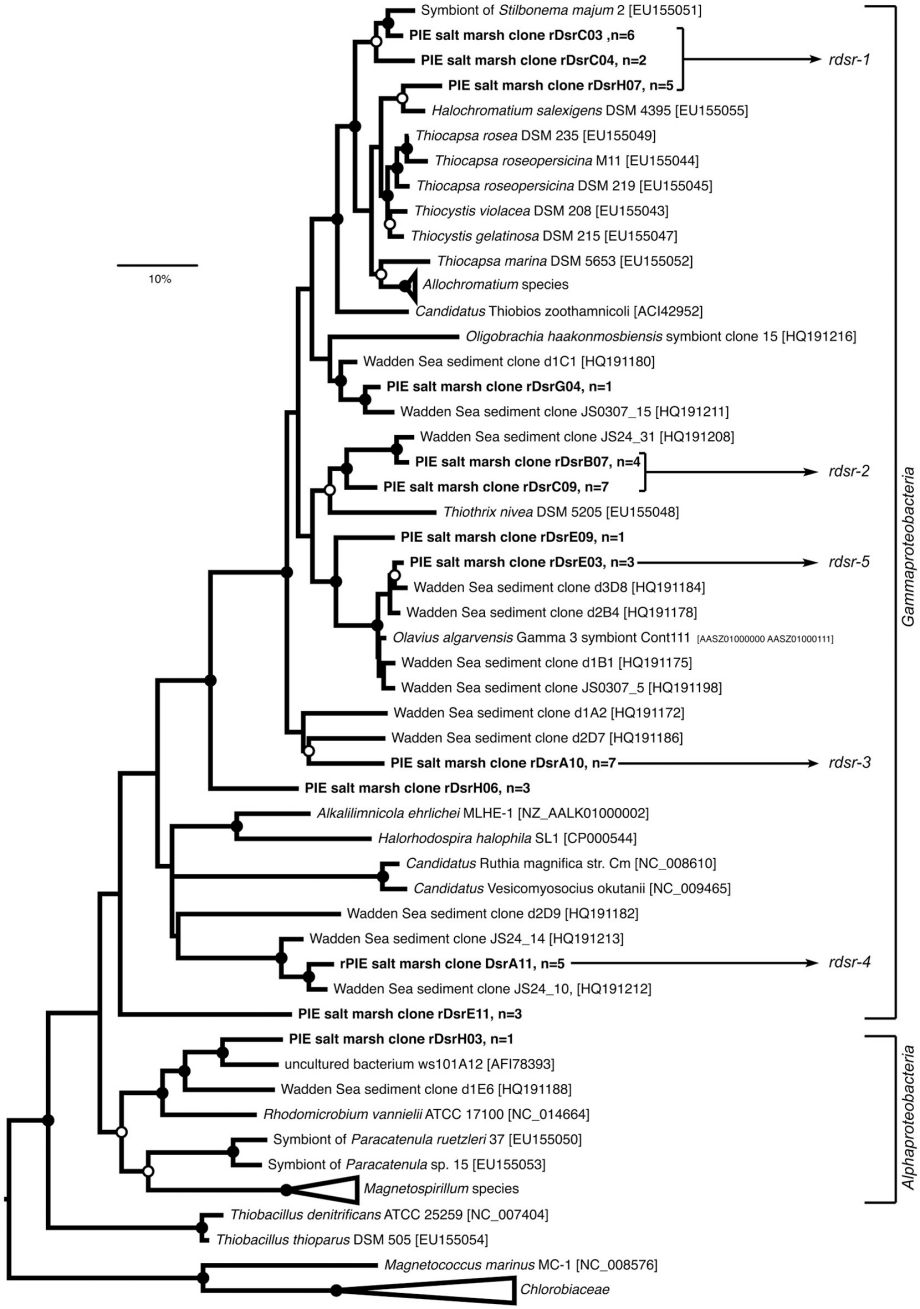
**Maximum-likelihood (ML) phylogenetic reconstruction of rDsrAB proteins deduced from sequences cloned from Plum Island Estuary salt marsh sediments (in boldface), including publicly available rDsrAB sequences from reference strains and uncultured bacteria (accession numbers are given)**. The WAG+G substitution model was used (100 re-samplings, *G* = 1.17, *I* = 0.19) based on testing different models in MEGA5. OTUs defined at 90% identity threshold are represented by one selected clone; “*n*” equals number of sequences per OTU. ML bootstrap support (100 resamplings) greater than 50% (open circles) and 70% (black circles) are displayed. Sequences from *Magnetococcus marinus* and *Chlorobiaceae* were used as outgroups. The bar indicates 10% sequence divergence. OTUs used to design *rdsr*AB primer sets targeting selected phylotypes for qPCR are shown (*rdsr* 1–5, Table Supp1).

*rdsr*AB sequences appeared to be less diverse than *sox*B. The clone library comprised 48 sequences representing 13 OTUs (Figure 2), almost fully covering the diversity as suggested by rarefaction analysis (Figure Supp1). All except one sequence clustered with *Gammaproteobacteria*, mirroring the known distribution of the rDSR pathway, which is mostly present in this class.

### Depth profile of s-oxidizer genes and transcripts

Using the “mix” samples collected in July at Site 2, we estimated the variations in abundance (i.e., DNA level) and transcriptional activity (i.e., RNA level) of S-oxidizers from surface to 20 cm using two methods: 16S rRNA amplicon tag sequencing, and newly designed qPCR assays of selected *sox*B and *rdsr*AB phylotypes.

**Figure 3:**
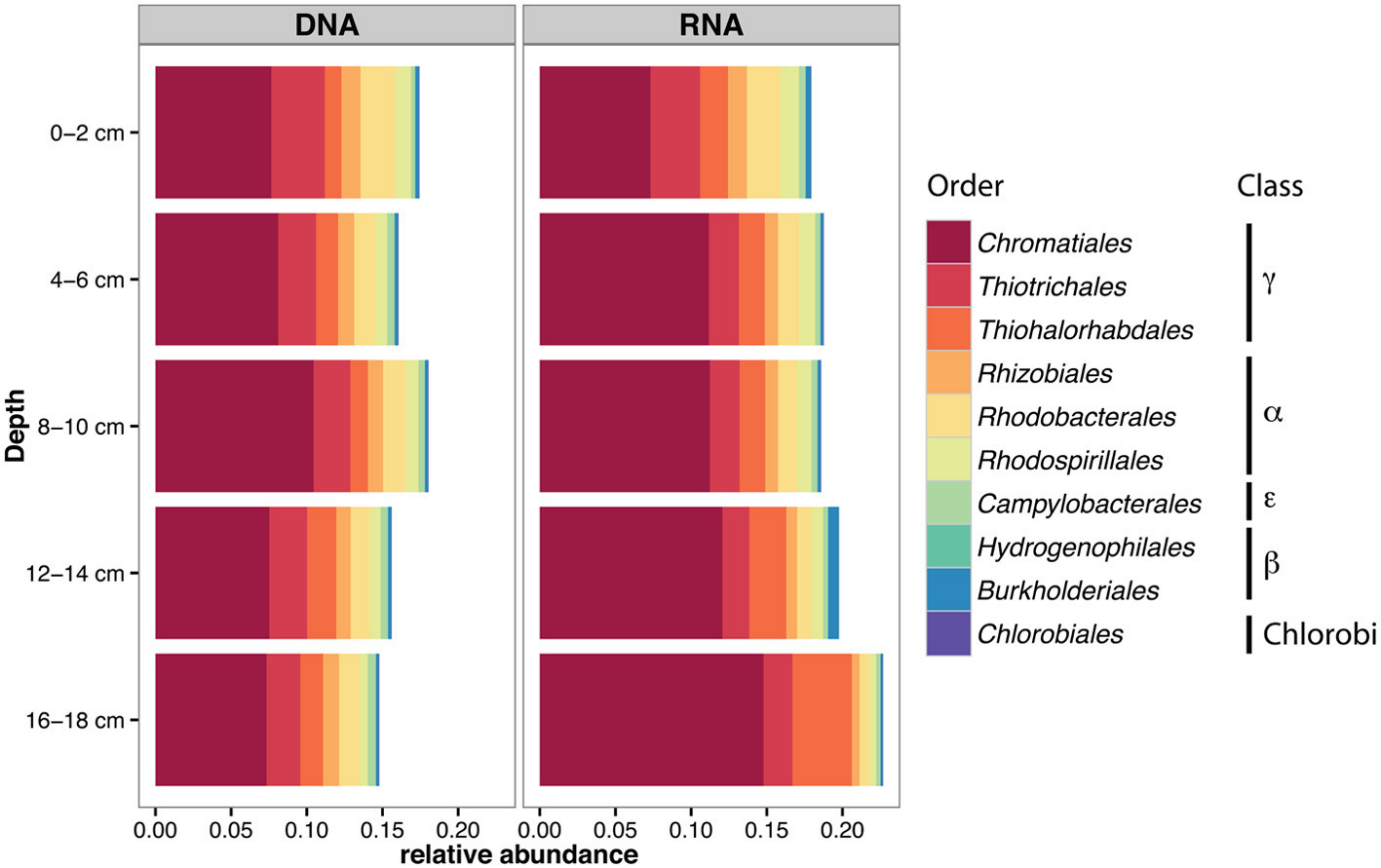
**Relative abundance of bacterial orders comprising S-oxidizers in 16S rRNA amplicon libraries from different depths in July at Site 2, in the DNA and RNA fractions**.

Based on bacterial 16S reads at the DNA level, the relative abundance of the orders containing potential S-oxidizers was stable along the depth profile (Figure 3), with *Chromatiales* and *Thiotrichales* the dominant groups. At the RNA level, the contribution of *Chromatiales* increased with depth, from 7% at the sediment surface to 15% between 16 and 18 cm (Spearman's rank correlation coefficient ρ = 1.00, *P* = 0.017), whereas the contribution of the three alphaproteobacterial orders *Rhizobiales, Rhodobacterales* and *Rhodospirillales* decreased with depth (ρ = −1.00, *P* < 0.02 for all 3 orders).

**Figure 4:**
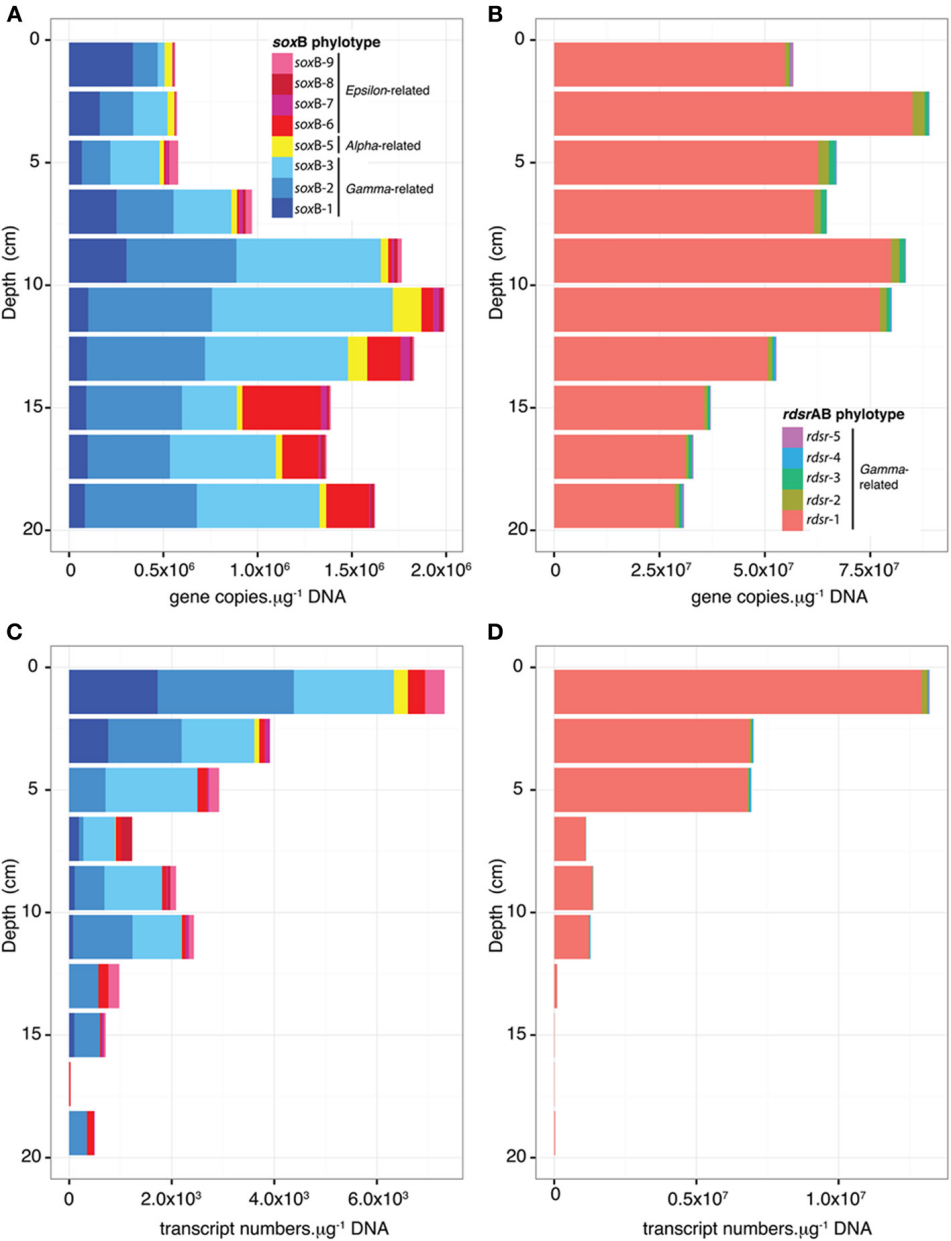
**Variations in gene (A,B) and transcript (C,D) abundance for *sox*B (A,C) and *rdsr*AB phylotypes (B,D) as a function of depth in July at Site 2**. Copy numbers were calculated using standard curve and efficiency as reported in Table Supp2.

qPCR assays showed that the distribution of functional gene copies with sediment depth does not match the distribution of transcripts (Figure 4). For *sox*B, the total copy numbers at the DNA level was positively correlated with depth (ρ = 0.65, *P* = 0.049, Figure 4A). Together, the *Thiotrichales*-related phylotypes *sox*B-2 and *sox*B-3 were the most abundant at all depths except at the surface where the *Congregibacter*-related *sox*B-1 contributed 60% of the total gene copies measured. Below 14 cm, the abundance of *Epsilonproteobacteria*-related *sox*B-6 increased. Positive correlations between copy numbers of individual phylotypes and sediment depth were detected for *sox*B-2 (ρ = 0.70, *P* = 0.031), *sox*B-6 (ρ = 0.96, *P* < 0.001) and *sox*B-8 (ρ = 0.73, *P* = 0.021). At the RNA level (Figure 4C), there was a strong decline with depth in total *sox*B transcript numbers (ρ = −0.94, *P* < 0.001). Significant negative correlations were found for the phylotypes *sox*B-1 to *sox*B-5, but not for the *Epsilonproteobacteria*-related *sox*B-6 to *sox*B-9. Similar to what was observed at the DNA level, transcripts of the *Thiothrix*-related *sox*B-2 and *sox*B-3 were the most detected *sox*B phylotypes.

For *rdsr*AB phylotypes, the total copy number was 1–3 orders of magnitude higher than that of *sox*B in all samples both at DNA and RNA levels (Figures 4B,D) and decreased with depth (DNA: ρ = −0.72, *P* = 0.024; RNA: ρ = −0.93, *P* < 0.001). Significant negative correlations (−0.95 < ρ < −0.71, *P* < 0.021) were found between sample depth and transcript numbers for all *rdsr*AB phylotypes. The *Chromatiales*-related phylotype *rdsr*1 was by far the most abundant and expressed.

### Effect of site and small-scale compartments on s-oxidizer communities

**Figure 5:**
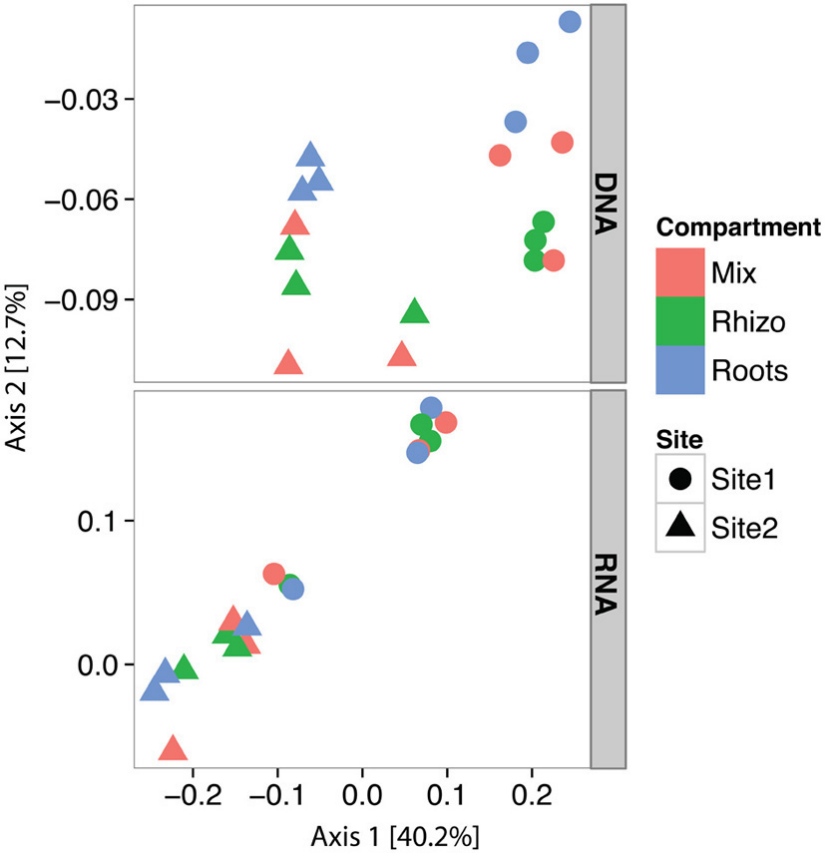
**Principal Coordinates Analysis (PCoA) plots of the potential S-oxidizer community composition in relation to nucleic acid, sampling site and small-scale compartment sampled**. PCoA ordination was performed on 16S rRNA amplicon sequence data for DNA and cDNA together, but displayed in distinct panels for clarity. All OTUs affiliated to orders listed in Table 1 were used.

**Figure 6:**
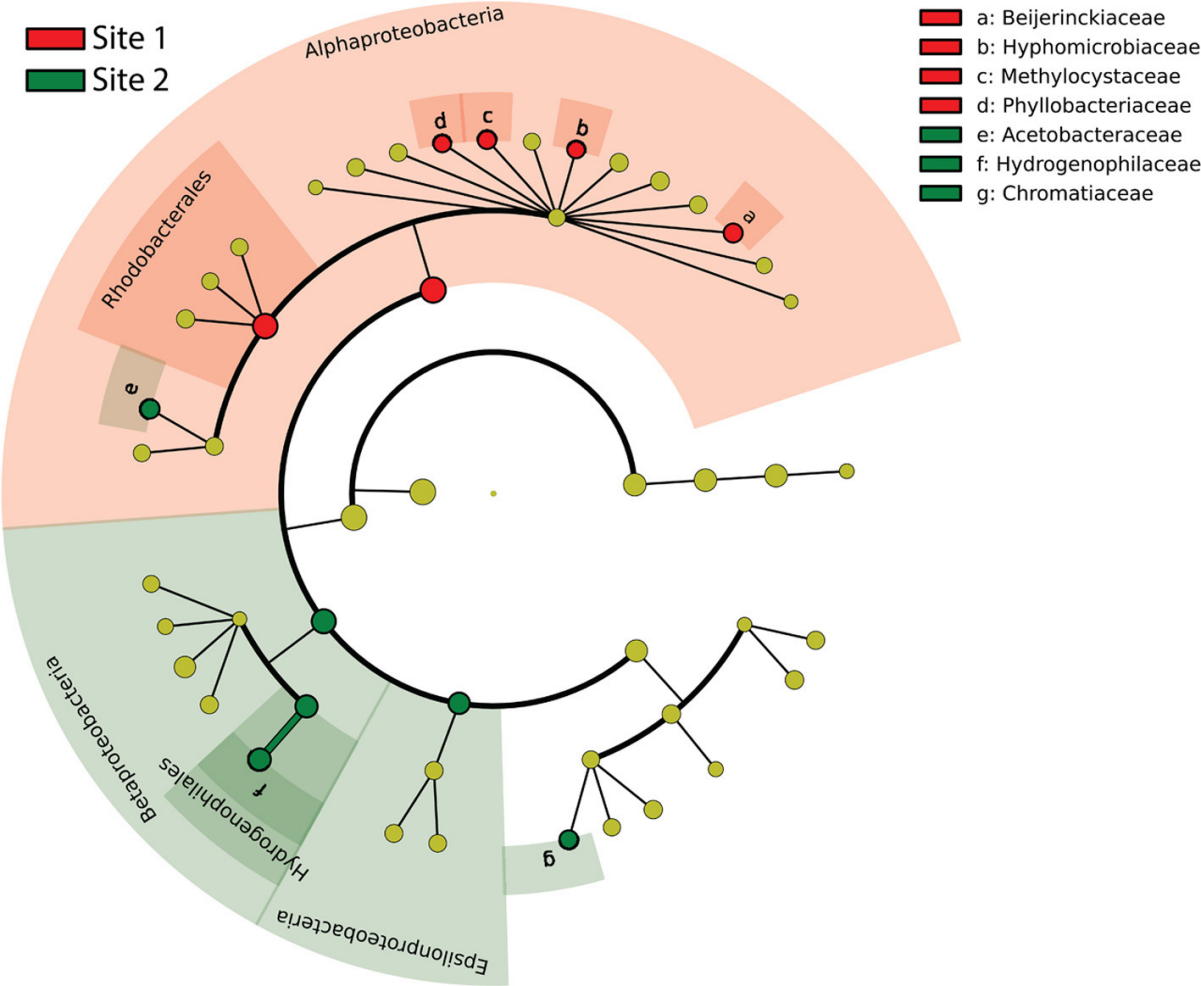
**Cladogram indicating the taxonomic distribution of potential S-oxidizer lineages statistically different between sampling sites, based on 16S rRNA amplicon sequence data**. Lineages with LDA 2.0 or higher determined by LEfSe are displayed. Red circles and shading indicate lineages enriched at Site 1; green circles and shading indicate lineages enriched at Site 2. Yellow circles denote non-significantly different lineages.

Samples collected in October at 5 cm depth were used to investigate the variations in S-oxidizer community composition, abundance and transcriptional activity at two sites and in three different compartments (mix, rhizosphere and roots). PCoA of potential S-oxidizers 16S rRNA OTUs (Figure 5) showed that the composition of the community differed strongly according to sampling site, both at the DNA level [PERMANOVA, *F*_(1, 16)_ = 13.27, *P* < 0.001, *R*^2^ = 0.45] and RNA level [PERMANOVA, *F*_(1, 16)_ = 11.38, *P* < 0.001, *R*^2^ = 0.42]. This was mainly due to enrichment in *Alphaproteobacteria* at Site 1 and in *Chromatiaceae, Beta*- and *Epsilon proteobacteria* at Site 2, as revealed by the LEfSe analysis (Figure 6). In addition, there was a significant effect of the compartment within Site 1 and Site 2 at the DNA level [PERMANOVA, *F*_(2, 15)_ = 0.95, *P* = 0.008, *R*^2^ = 0.112], where communities in “roots” samples tended to cluster away from rhizosphere samples (Figure 5, top panel).

**Figure 7:**
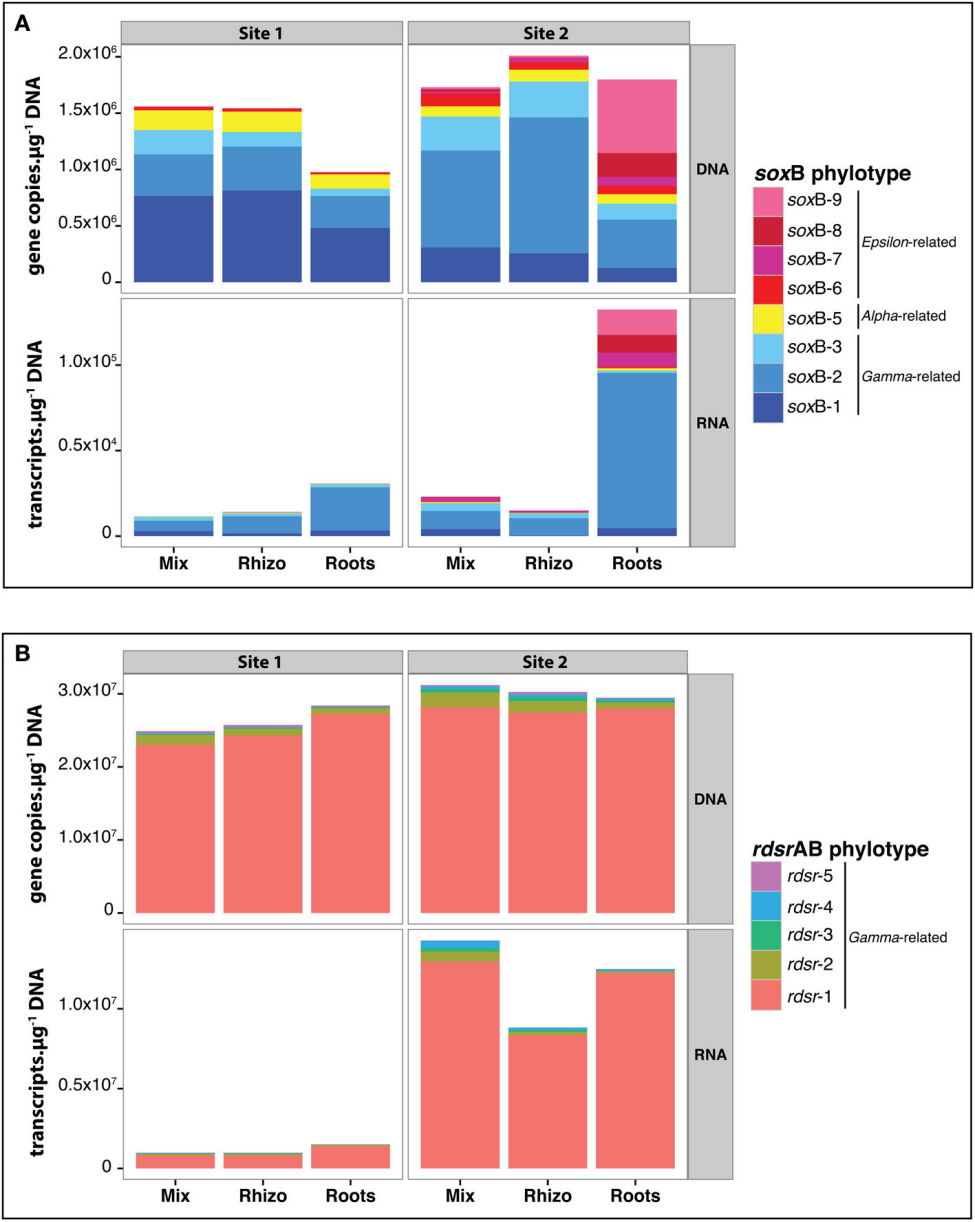
**Variations in gene and transcript abundance for *sox*B (A) and *rdsr*AB phylotypes (B) for samples at 5 cm depth from the two sites in October**. Values are mean copy numbers (*n* = 3). Copy numbers were calculated using standard curves and efficiencies reported in Table Supp2. Details (including standard errors) for each phylotype are given in Figures Supp2–Supp5.

In general, total *rdsr*AB gene and transcripts numbers (Figure 7B) were 1–3 orders of magnitude higher than those of *sox*B (Figure 7A). In all three compartments, *Chromatiales* and *Thiotrichales* represented the most abundant and expressed phylotypes for *rdsr*AB (*rdsr*-1) and *sox*B (*sox*B-2 and 3), respectively (Figure 7). No differences between the mix and rhizosphere samples were found for the copy and transcript numbers of any *sox*B or *rdsr*AB phylotype (Figures Supp2–Supp5). In contrast, significant differences were observed between roots samples and at least one other compartment for *Gamma*- and *Epsilonproteobacteria* phylotypes.

Gene copy abundances for the *Gammaproteobacteria*-related *sox*B phylotypes (*sox*B-1, -2, and -3) were lower in roots samples than in mix samples at Site 2 (Figure Supp2). At Site 1, the same trend was apparent but the pattern was significant only for *sox*B-3. No significant differences were found at the transcript level (Figure Supp3). For the five *Gammaproteobacteria*-related *rdsrAB* phylotypes, no significant differences in gene copy abundance were found in roots vs. mix samples (Figure Supp4), but three of the five phylotypes (*rdsr*-2, -3, and -4) showed decreased transcript abundance in roots samples compared to mix samples at Site 2 (Figure Supp5).

Gene copy and transcript numbers of *Epsilonproteobacteria sox*B-phylotypes (*sox*B-6, -7, -8- and -9) were much higher at Site 2, in line with the 16S rRNA data. At this site, their contribution tended to increase on *Spartina* roots samples compared to the mix samples (Figure Supp2), and the pattern for *sox*B-7, and *sox*B-9 was significant. Transcripts for all *Epsilonproteobacteria*-related *sox*B phylotypes tended to be present in higher numbers in roots samples (compared to mix and rhizosphere) at Site 2 (Figure Supp3), though small sample size (*n* = 3) and high variability precluded statistical significance (e.g., for *sox*B-9, *P* = 0.09).

## Discussion

### *Chromatiales* and *thiotrichales* are dominant s-oxidizers in salt marsh sediments

16S rRNA tag sequencing, phylogenetic analyses of *sox*B and *rdsr*AB genes, as well as quantification of their copy and transcript numbers congruently showed that *Gammaproteobacteria*, especially members of the orders *Chromatiales* and *Thiotrichales*, are the dominant S-oxidizers in salt marsh sediments populated with *S. alterniflora*. S-oxidizing members of the *Alpha*- and *Epsilonproteobacteria* classes were also detected, although in lower numbers. The dominant taxa were only distantly related to cultivated strains of S-oxidizers and mainly grouped with uncultured environmental bacteria, underlining the lack of knowledge on sulfur-oxidation in salt marshes.

Dominance of gammaproteobacterial S-oxidizers was also found in unvegetated intertidal sediments of the German Wadden Sea, mostly belonging to uncultured clades or related to *Thiothrix nivea* (Lenk et al., 2011). In addition, we identified organisms related to *Congregibacter litoralis* belonging to the NOR5/OM60 clade, also detected in Wadden Sea sediments (Lenk et al., 2011, 2012). Interestingly, we show that the phylotype belonging to this clade (*sox*B-1) is transcribed in surface salt marsh sediments, even though evidence for thiotrophy is currently lacking for this clade (Fuchs et al., 2007; Lenk et al., 2012). S-oxidizers belonging to *Chromatiales* were identified as an important group in our study, while they were absent in the Wadden Sea sediments (Lenk et al., 2011). Most of the epsilonproteobacterial S-oxidizers we identified in the salt marsh sediments were related to *Sulfurovum* and *Sulfurimonas*, similar to those found in other reduced marine environments, including tidal mud flats (Timmer-Ten Hoor, 1975), pelagic redoxclines (e.g., Grote et al., 2008; Bruckner et al., 2012), cold seeps (e.g., Niemann et al., 2013) and hydrothermal vents (e.g., Hügler et al., 2010; Akerman et al., 2013). What might make this particular group of S-oxidizers successful in these different habitats is an outstanding question.

Our study is the first to quantify *rdsr*AB and *sox*B genes simultaneously in the environment. The higher copy numbers of *rdsr*AB compared to *sox*B was unexpected, as bacteria featuring the reverse-DSR pathway typically possess both genes and use the SOX system to oxidize thiosulfate (Kappler and Dahl, 2001). This discrepancy could be explained by the fact that we were unable to design specific primers for some potentially abundant *sox*B phylotypes (e.g., targeting GenE01 or GenB02, Figure 1). However, as *rdsr*AB genes are usually present in only one copy per genome (Loy et al., 2009), this could also point to some S-oxidizers using the reverse-DSR pathway either possessing a divergent SOX system or lacking it, in which case they might be unable to utilize thiosulfate (Petri et al., 2001; Meyer et al., 2007).

### Distribution of *soxb* and *rdsrAB* gene copies does not match the distribution of transcripts with depth

Our data suggest that the transcriptional activity of the S-oxidizers occurs mainly in the upper 5 cm, largely following the expected distribution of root biomass (Davey et al., 2011). The decrease in transcription in deeper sediments may be due to a depletion of potential electron acceptors such as oxygen or nitrate, which are both influenced by the roots, either directly in the case of oxygen (via provision of O_2_ through aerenchyma or use of O_2_ for respiration), or indirectly via rhizosphere nitrification in the case of nitrate. In addition, inhibition by high sulfide concentration could occur (Ruby and Jannasch, 1982; Wirsen et al., 2002). Interestingly, transcripts of the *Thiotrichales*-affiliated phylotypes *sox*B-2 and *sox*B-3 were still detected below 10 cm, but the *rdsr*-2 phylotype (also affiliated to *Thiotrichales*) was barely expressed (Figures 4C,D). For these organisms, this transcriptional uncoupling between *sox*B and *rdsr*AB could be due to a lack of terminal electron acceptors, which would prevent the full oxidation of reduced sulfur compounds all the way to sulfate. This might lead to the accumulation of elemental sulfur as an intermediate due to a repression of *rdsr*AB, which is involved in the remobilisation of sulfur globules as the first step to complete the oxidation to sulfate.

Overall, the abundance of *sox*B genes from *Epsilonproteobacteria* (*sox*B-6 to *sox*B-9) increased with depth (Figure 4A), but transcript numbers did not. However, because *sox*B-1, *sox*B-2, and *sox*B-3 transcript numbers plummeted with depth, the relative abundance of *sox*B-6 to *sox*B-9 transcripts increased, accounting for about 30% of the detected *sox*B transcripts below 12 cm. So far all characterized sulfur-oxidizing *Epsilonproteobacteria* lack the reverse-DSR pathway, therefore relying on the SOX pathway for S-oxidation. As discussed above, *sox*B-2 and *sox*B-3 transcripts from *Thiotrichales* detected below 10 cm may reflect partial S-oxidation to elemental sulfur. Although found in low absolute abundance, *Epsilonproteobacteria* may thus account for a significant portion of S-oxidation in deeper salt marsh sediments, perhaps enabled by their association with *Spartina* roots and their adaptation to microaerobic conditions (see below).

### Microbial community membership and transcriptional activity vary between sites 1 and 2 though both are vegetated with *s. alterniflora*

We observed pronounced differences in S-oxidizer communities between the two sampling locations, even though both were vegetated with the same plant. The transcription of S-oxidizing genes was generally lower at Site 1. Furthermore, *Epsilonproteobacteria* and *Chromatiales*-related phylotypes were present in higher abundance at Site 2 (Figures 6, 7, Supp2, Supp3). In contrast, *Alphaproteobacteria*-related S-oxidizers were found in higher abundance at Site 1 based on the 16S rRNA libraries (Figure 6). Some of these patterns may relate to different salinity and flooding conditions. Site 2, which is closer to the mouth of the estuary and away from the creekbank, has a higher salinity and less frequent tidal flushing. Samples from Site 1 were collected directly at the creekbank, which based on previous work is characterized by lower sulfate reduction rates as compared to interior marsh sites, likely due to more extensive porewater drainage and air entry favoring aerobic decomposition (Howarth and Giblin, 1983; King, 1988). In addition, more frequent and longer tidal flushing may prevent the accumulation of sulfide at Site 1. Indeed, subsequent measurements obtained in July 2013 for porewater at 5 cm depth showed higher concentrations of sulfide at Site 2 (0.7–1.6 mM) than at Site 1 (0–0.01 mM, unpublished). Thus, conditions at Site 2 are likely to favor *Epsilonproteobacteria*, which have predominantly been identified from sulfidic marine environments, and are generally known to tolerate higher sulfide concentrations (Wirsen et al., 2002; Campbell et al., 2006).

### Evidence for small-scale niche structure in the rhizosphere

At the DNA level, our results showed a significant effect of the compartment on the composition of S-oxidizer communities based on 16S rRNA tags, with roots samples differing from rhizosphere (Figure 5). Interestingly, the same effect was not observed at the RNA level, suggesting that though active community composition varies among sites and in roots relative to surrounding sediments, a common pool of S-oxidizers is active across compartments. Marker genes for S-oxidation were detected in the DNA and RNA fractions of mix, rhizosphere and roots samples. This suggests that S-oxidizers grow and are active in all compartments, although the expression of fully functional S-oxidation pathways would need to be confirmed at the protein level. We further observed distinct patterns in gene and transcript numbers of *sox*B and *rdsr*AB phylotypes on the roots. Therefore, the *S. alterniflora* root environment actually influences S-oxidizers from a common pool of species by fine-tuning their abundance and transcriptional activity. In particular, the global expression of *sox*B was higher at both sites on the roots compared to mix and rhizosphere samples (Figure 7), suggesting that the root surface environment might favor the activity of some S-oxidizers in vegetated sediments. They might benefit from the localized production of sulfide by root-associated sulfate-reducers (Hines et al., 1999) and the release of oxygen from the roots, enabling their activity in deeper sediments. In addition, denitrifying S-oxidizers would also be able to use nitrate formed around the roots by nitrification (Hamersley and Howes, 2005).

Most of the *rdsr*AB and *sox*B transcripts detected on the roots belonged to *Gammaproteobacteria*, largely dominated by *rdsr*-1 and *sox*B-2, respectively. However, we found an interesting pattern for *Epsilonproteobacteria* at Site 2, where this class was enriched. Although *Epsilonproteobacteria* were only a minor fraction of the *sox*B phylotypes detected in mix and rhizosphere samples, they accounted on average for 56 and 26% of the *sox*B copies and transcripts detected on roots, respectively. This suggests that the root system of *S. alterniflora* might constitute a preferential niche for the establishment of sulfur-oxidizing *Epsilonproteobacteria*, compared to the surrounding sediment. This finding was not expected considering that cultivated S-oxidizing *Epsilonproteobacteria* are known to be microaerophiles and to use oxygen-sensitive enzymes for carbon fixation (Hügler and Sievert, 2011). Further work to characterize the conditions in the rhizoplane in more detail and/or isolation and characterization of sulfur-oxidizing *Epsilonproteobacteria* from root surfaces is required to solve this apparent paradox.

Previous studies reported the capacity of *S. alterniflora* root tips to oxidize sulfide via both enzymatic and non-enzymatic mechanisms (Lee, 1999). To our knowledge, this is the first identification of specific bacterial groups on *S. alterniflora* roots that could contribute to S-oxidation. The present study complements the existing knowledge on the sulfur cycle in salt marshes by providing lacking information on its oxidative part, setting the stage for future investigations exploring the effect of environmental conditions and plant-microorganism interactions in more detail. In particular, we showed the expression of genes for two S-oxidation pathways up to the transcript level, which would need to be supplemented with proteomic studies and rate measurements. Furthermore, the evidence for small-scale heterogeneity of S-oxidizers in the rhizosphere underlines the need to develop new biogeochemical techniques enabling non-destructive measurements of chemical concentrations and process rates *in situ* at the required sub-millimeter level.

### Conflict of interest statement

The authors declare that the research was conducted in the absence of any commercial or financial relationships that could be construed as a potential conflict of interest.
