# Supplementary material for: Rhizosphere heterogeneity shapes abundance and activity of sulfur-oxidizing bacteria in vegetated salt marsh sediments
Source: Front Microbiol. 2014 Jun 24;5:309. doi: 10.3389/fmicb.2014.00309 (PMC4068000; doi:10.3389/fmicb.2014.00309)
Supplement: Supplementary file 1 [file Presentation1.ZIP › Presentation 1(1)/Supplementary_tables.docx]

**Table Supp1.** Primers used for (RT)-qPCR. A suite of primers was designed to target 8 selected *sox*B phylotypes (4 affiliated with *Epsilon*-, 3 with *Gamma*- and 1 with *Alpha-proteobacteria*) and 5 *rdsr*AB *Gammaproteobacteria*-related phylotypes (Figures 4 and 5). Based on the number of clones obtained in the libraries, this represents 65% and 81% of the retrieved *sox*B and *rdsr*AB sequences, respectively.

| Phylotype | Amplicon size (bp) | Standard template | Primer | | |
| --- | --- | --- | --- | --- | --- |
|  |  |  | Name | Sequence (5’→3’) | %GC |
| soxB-1 | 90 | general_C04 | soxB-1f | CCG ACG ATG CCA TCT T | 56.2 |
|  |  |  | soxB-1r | GAC CGC CGA CTT CTC | 66.7 |
|  |  |  |  |  |  |
| soxB-2 | 75 | general_A02 | soxB-2f | TTA TGG TGC CGG ACT G | 56.2 |
|  |  |  | soxB-2r | CCT TAC CGC GTG CTT | 60.0 |
|  |  |  |  |  |  |
| soxB-3 | 117 | general_C08 | soxB-3f | GCC AAT CCT CGC TAC A | 56.2 |
|  |  |  | soxB-3r | GTG TGA CAG CAC GAT AAC | 50.0 |
|  |  |  |  |  |  |
| soxB-5 | 83 | general_B08 | soxB-5f | GGC TAC TCG TCC AAC C | 62.5 |
|  |  |  | soxB-5r | CGC ACC TCG TCA ATC T | 56.2 |
|  |  |  |  |  |  |
| soxB-6 | 185 | Epsilon_C04 | soxB-6f | TCC CAC CAT ACT CTA TCA AAG | 42.9 |
|  |  |  | soxB-6r | AAC GAC TGC ATC CAC TT | 47.1 |
|  |  |  |  |  |  |
| soxB-7 | 108 | Epsilon_A08 | soxB-7f | TCA TCG GGC AGT CCT | 60.0 |
|  |  |  | soxB-7r | GCT CGT TGA CAT ACT CCT | 50.0 |
|  |  |  |  |  |  |
| soxB-8 | 155 | Epsilon_B03 | soxB-8f | TTA CGG AAG GTT GGA GTT | 44.4 |
|  |  |  | soxB-8r | GTC AAT ACC ATG TAC CAT ACG | 42.9 |
|  |  |  |  |  |  |
| soxB-9 | 143 | Epsilon_D12 | soxB-9f | TCR CCA AAG CCT ATT ACT ATT G | 36.4-40.9 |
|  |  |  | soxB-9r | ATA TTT GAK GCA ATC GGA AT | 30.0-35.0 |
|  |  |  |  |  |  |
| rdsr-1 | 154 | DsrH07 | rdsr-1f | TGC GGM GAR ATG ATC | 46.7-60.0 |
|  |  |  | rdsr-1r | CYT GRC GCT CGA ACC | 60.0-73.3 |
|  |  |  |  |  |  |
| rdsr-2 | 91 | DsrB07 | rdsr-2f | GAA TTT CCT CGA AGG CAT C | 47.4 |
|  |  |  | rdsr-2r | GTC CCA GCC ATC CAT G | 62.5 |
|  |  |  |  |  |  |
| rdsr-3 | 86 | DsrA10 | rdsr-3f | GCG TGG TCA AGG CTA T | 56.2 |
|  |  |  | rdsr-3r | GCA AGA AGT GGT GAT ATG G | 47.4 |
|  |  |  |  |  |  |
| rdsr-4 | 108 | DsrA11 | rdsr-4f | GAT ACG GTG GCG GAA T | 56.2 |
|  |  |  | rdsr-4r | CTG GAG GTG GCA TTA CA | 52.9 |
|  |  |  |  |  |  |
| rdsr-5 | 99 | DsrE03 | rdsr-5f | GCC TGG TCA CCT TCC | 66.7 |
|  |  |  | rdsr-5r | AGC CGT ACT CGT TGA TT | 47.1 |
| Design parameters were as follows: primer T_m_: 59ºC to 61ºC; length: 15 to 25 bp; run/repeat max. length = 3; GC clamp = 1; amplicon size: 75 to 200 bp. | | | | | |

**Table Supp2.** Conditions and performances of the qPCR assays.

| Phylotype | Primer concn f/r (µM) | Annealing temp (ºC) | qPCR performance | | | | |
| --- | --- | --- | --- | --- | --- | --- | --- |
|  |  |  | r^2^ | y intercept | efficiency (%) | C_T_ cutoff | Linear dynamic  range |
| soxB-1 | 0.9 / 0.3 | 60 | 0.999 | 35.66 | 81.4 | NTC undetected | 10-10^5^ copies |
| soxB-2 | 0.3 / 0.3 | 60 | 0.999 | 35.44 | 82.5 | NTC undetected | 10-10^5^ copies |
| soxB-3 | 0.3 / 0.3 | 60 | 0.994 | 32.61 | 89.9 | NTC undetected | 10-10^5^ copies |
| soxB-5 | 0.3 / 0.3 | 55 | 0.999 | 32.82 | 95.7 | NTC undetected | 10-10^5^ copies |
| soxB-6 | 0.5 / 0.5 | 55 | 0.999 | 35.10 | 86.8 | 33.23 | 10-10^5^ copies |
| soxB-7 | 0.3 / 0.3 | 55 | 0.995 | 32.45 | 90.0 | NTC undetected | 10-10^5^ copies |
| soxB-8 | 0.5 / 0.5 | 55 | 0.998 | 32.46 | 92.9 | NTC undetected | 10-10^5^ copies |
| soxB-9 | 0.5^a^ / 0.5^a^ | 55 | 0.999 | 33.34 | 93.3 | 33.48 | 10-10^5^ copies |
| rdsr-1 | 0.5^a^ / 0.5^a^ | 58 | 0.994 | 38.41 | 81.6 | 32.06 | 10-10^5^ copies |
| rdsr-2 | 0.5 / 0.5 | 60 | 0.998 | 33.07 | 94.0 | NTC undetected | 10-10^5^ copies |
| rdsr-3 | 0.5 / 0.5 | 55 | 0.998 | 32.40 | 96.5 | NTC undetected | 10-10^5^ copies |
| rdsr-4 | 0.5 / 0.5 | 60 | 0.999 | 33.47 | 90.6 | NTC undetected | 10-10^5^ copies |
| rdsr-5 | 0.5 / 0.5 | 55 | 0.999 | 32.21 | 96.8 | NTC undetected | 10-10^5^ copies |
| ^a^ for degenerate primers, concentrations are for each individual non-degenerate oligonucleotide | | | | | | |  |

**Table Supp3**: Number of reads and OTUs obtained for each sample from the DNA and cDNA 16S rRNA gene amplicon libraries.

| Season | Site | Depth | Compartment | Replicate | DNA | | |  | RNA | | |
| --- | --- | --- | --- | --- | --- | --- | --- | --- | --- | --- | --- |
|  |  |  |  |  | Reads | OTUs^a^ | Rarefied  OTUs^b^ |  | Reads | OTUs^a^ | Rarefied  OTUs^b^ |
| July | Site 2 | 0-2 cm | Mix | 1 | 426054 | 22269 | 14187 |  | 142916 | 14578 | 14578 |
| July | Site 2 | 4-6 cm | Mix | 1 | 460655 | 21512 | 13099 |  | 221225 | 16566 | 13809 |
| July | Site 2 | 8-10 cm | Mix | 1 | 510283 | 22876 | 13450 |  | 261307 | 18024 | 13868 |
| July | Site 2 | 12-14 cm | Mix | 1 | 516468 | 23647 | 13940 |  | 271919 | 17332 | 13013 |
| July | Site 2 | 16-18 cm | Mix | 1 | 387296 | 20996 | 13804 |  | 347343 | 17194 | 11824 |
| October | Site 1 | 4-6 cm | Mix | 1 | 320798 | 18582 | 13063 |  | 184511 | 15925 | 14250 |
| October | Site 1 | 4-6 cm | Mix | 2 | 399237 | 20218 | 13057 |  | 214946 | 16897 | 14148 |
| October | Site 1 | 4-6 cm | Mix | 3 | 304651 | 17451 | 12765 |  | 168228 | 14983 | 13949 |
| October | Site 1 | 4-6 cm | Rhizosphere | 1 | 365758 | 19176 | 12729 |  | 176171 | 15677 | 14378 |
| October | Site 1 | 4-6 cm | Rhizosphere | 2 | 373414 | 20096 | 13344 |  | 250343 | 18392 | 14483 |
| October | Site 1 | 4-6 cm | Rhizosphere | 3 | 341110 | 18906 | 13090 |  | 189407 | 16083 | 14266 |
| October | Site 1 | 4-6 cm | Roots | 1 | 407544 | 18850 | 11896 |  | 148985 | 13957 | 13746 |
| October | Site 1 | 4-6 cm | Roots | 2 | 405760 | 18009 | 11502 |  | 228744 | 16813 | 13707 |
| October | Site 1 | 4-6 cm | Roots | 3 | 253770 | 16935 | 13211 |  | 199078 | 16100 | 14005 |
| October | Site 2 | 4-6 cm | Mix | 1 | 347554 | 19453 | 13142 |  | 274327 | 17692 | 13349 |
| October | Site 2 | 4-6 cm | Mix | 2 | 391105 | 21056 | 13810 |  | 243633 | 17557 | 13986 |
| October | Site 2 | 4-6 cm | Mix | 3 | 444795 | 21744 | 13496 |  | 171659 | 15045 | 13896 |
| October | Site 2 | 4-6 cm | Rhizosphere | 1 | 494044 | 22110 | 13158 |  | 211737 | 16227 | 13719 |
| October | Site 2 | 4-6 cm | Rhizosphere | 2 | 395746 | 20101 | 13097 |  | 216726 | 16548 | 13811 |
| October | Site 2 | 4-6 cm | Rhizosphere | 3 | 373005 | 20959 | 13949 |  | 167673 | 15180 | 14237 |
| October | Site 2 | 4-6 cm | Roots | 1 | 504445 | 22390 | 13235 |  | 234899 | 16036 | 12865 |
| October | Site 2 | 4-6 cm | Roots | 2 | 344707 | 19155 | 13061 |  | 259884 | 17051 | 13139 |
| October | Site 2 | 4-6 cm | Roots | 3 | 412861 | 20701 | 13028 |  | 207207 | 15653 | 13326 |
| ^a^ number of OTUs per sample before rarefaction to even depth.  ^b^ number of OTUs per sample after rarefaction to the lowest number of reads per sample (142916 reads). | | | | | | | | | | | |
