# Supplementary figures and images for: Rhizosphere heterogeneity shapes abundance and activity of sulfur-oxidizing bacteria in vegetated salt marsh sediments
Source: Front Microbiol. 2014 Jun 24;5:309. doi: 10.3389/fmicb.2014.00309 (PMC4068000; doi:10.3389/fmicb.2014.00309)

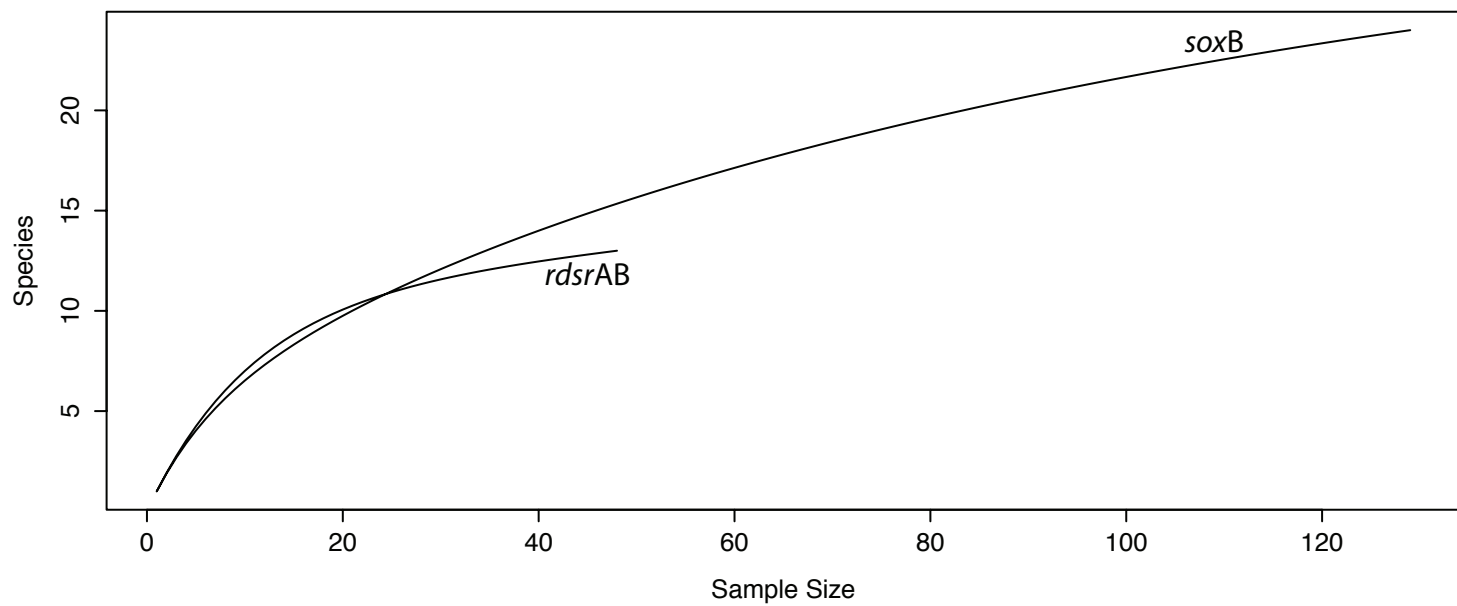

Supplement: Supplementary file 1 [file Presentation1.ZIP › Presentation 1(1)/FigureSupp1.pdf]

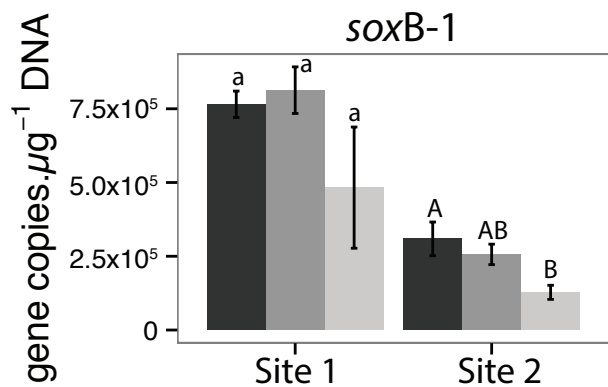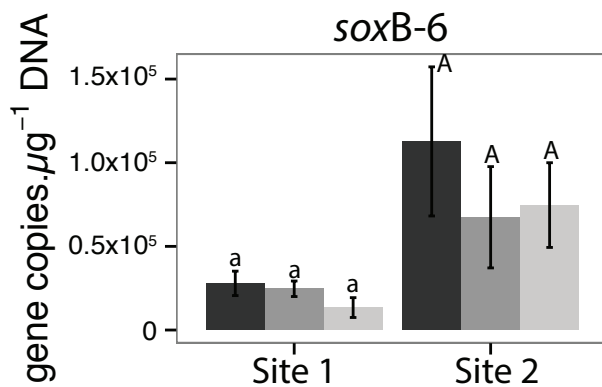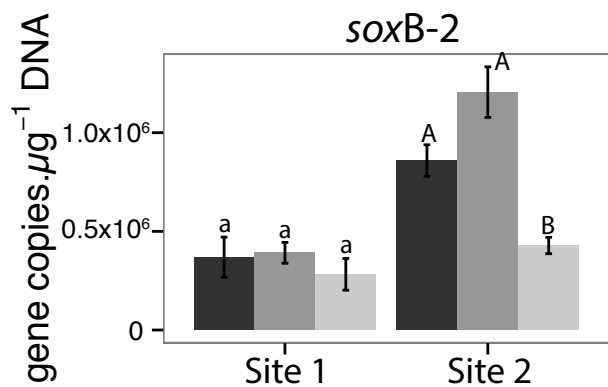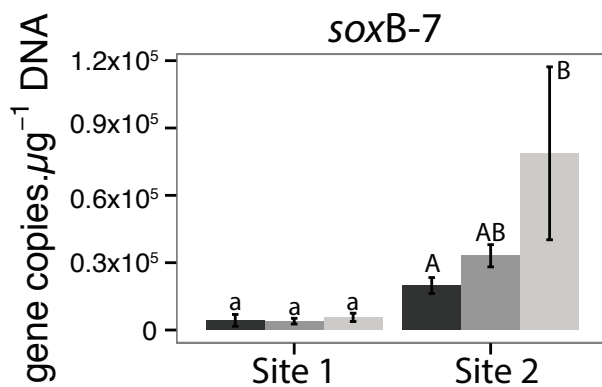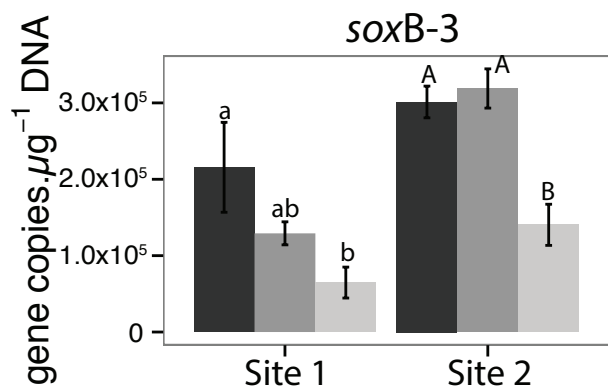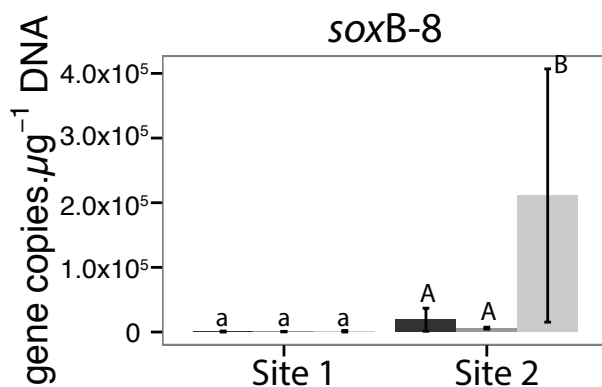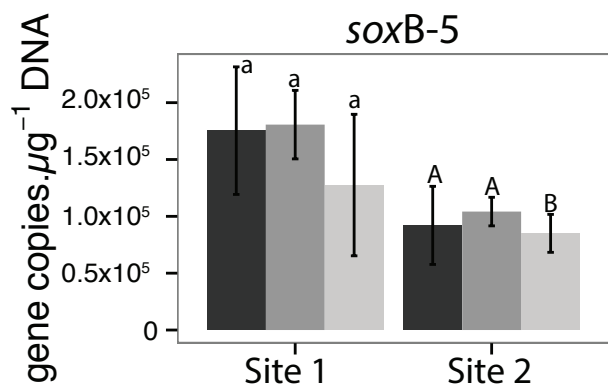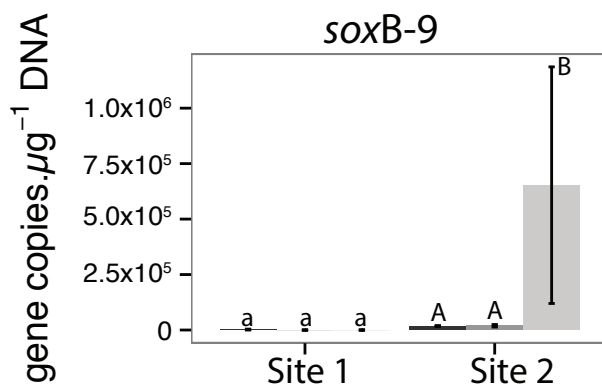

Supplement: Supplementary file 1 [file Presentation1.ZIP › Presentation 1(1)/FigureSupp2.pdf]

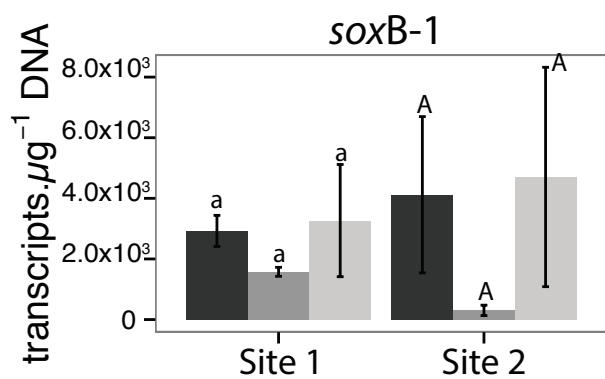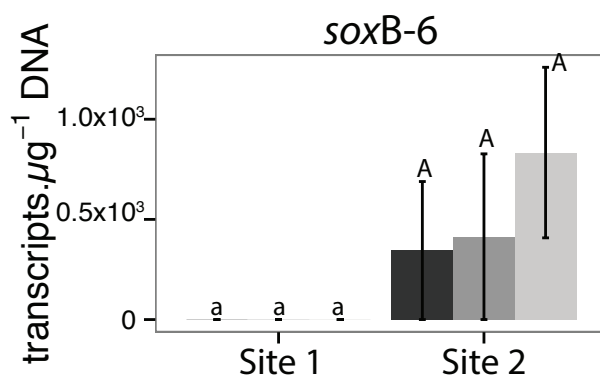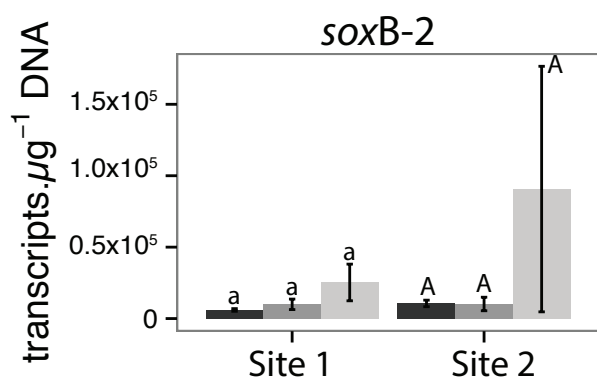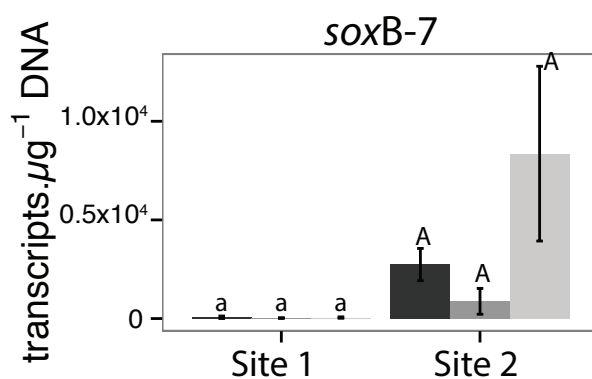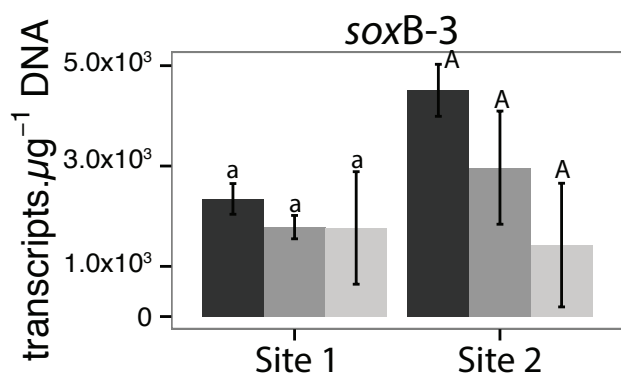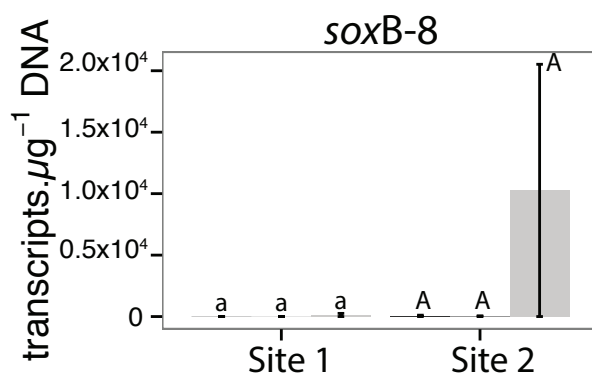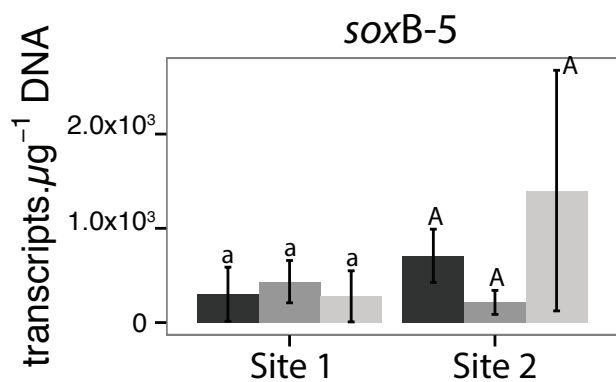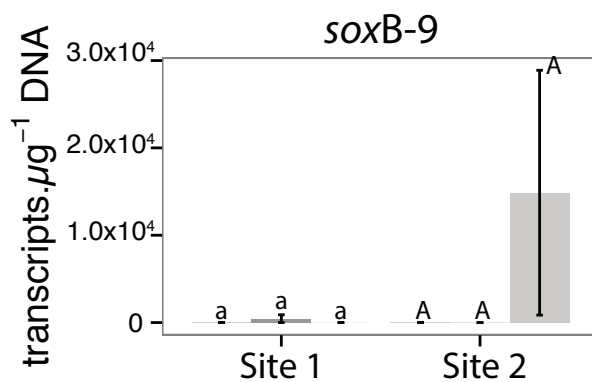

Supplement: Supplementary file 1 [file Presentation1.ZIP › Presentation 1(1)/FigureSupp3.pdf]

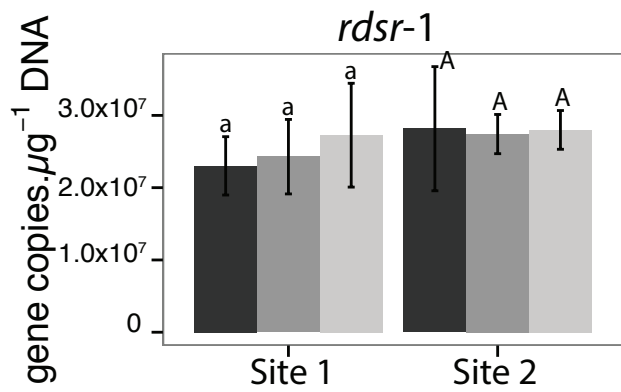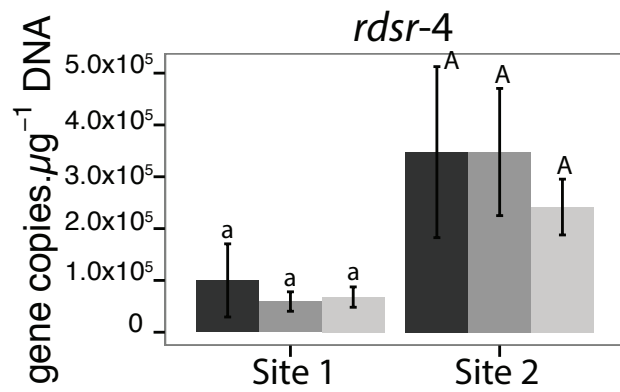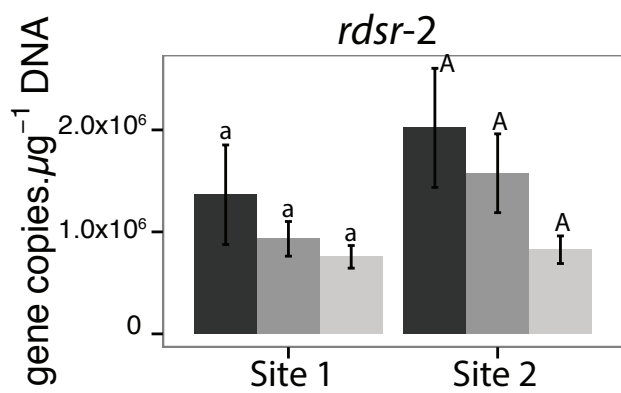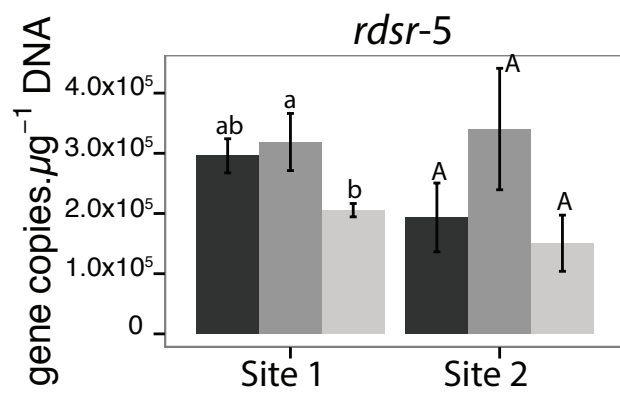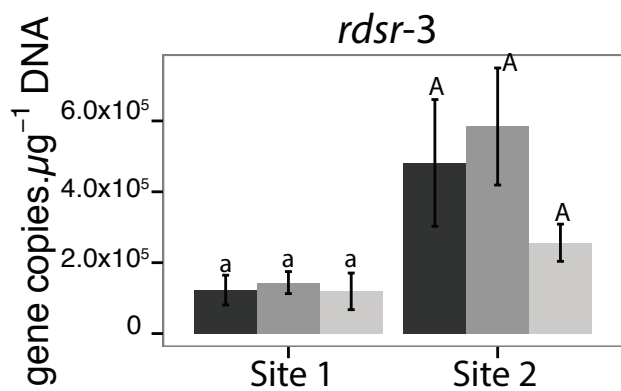

Supplement: Supplementary file 1 [file Presentation1.ZIP › Presentation 1(1)/FigureSupp4.pdf]

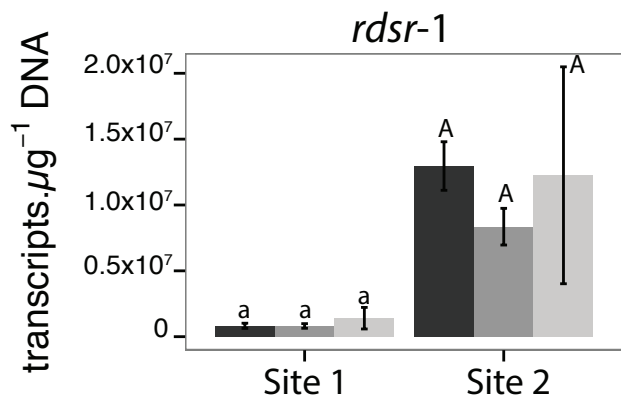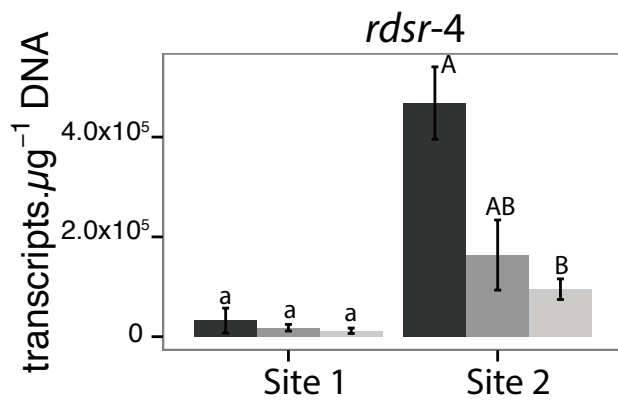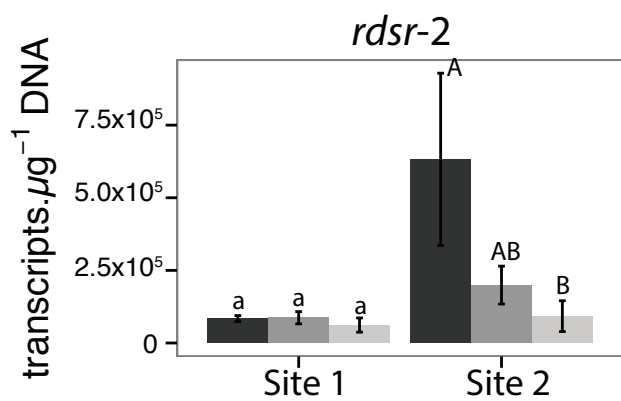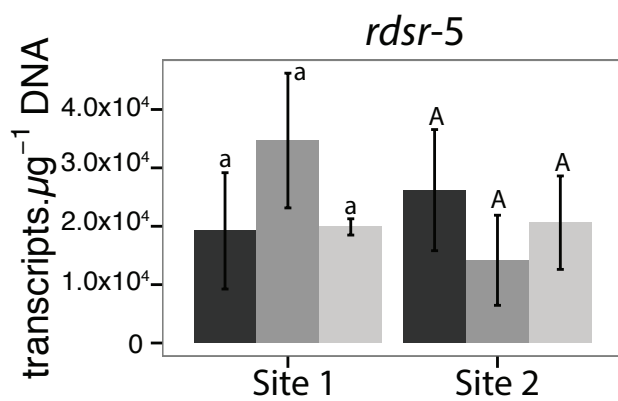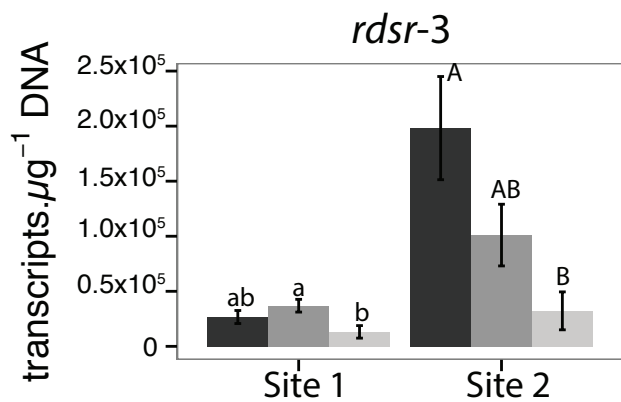

Supplement: Supplementary file 1 [file Presentation1.ZIP › Presentation 1(1)/FigureSupp5.pdf]
