# Supplementary material for: Rhizosphere heterogeneity shapes abundance and activity of sulfur-oxidizing bacteria in vegetated salt marsh sediments
Source: Front Microbiol. 2014 Jun 24;5:309. doi: 10.3389/fmicb.2014.00309 (PMC4068000; doi:10.3389/fmicb.2014.00309)
Supplement: Supplementary file 1 [file Presentation1.ZIP › Presentation 1(1)/Legends_to_Supplementary_Figures.doc]

**Figure Supp1:** Rarefaction curves of the OTUs number at 90% identity obtained from the *sox*B and *rdsr*AB clone libraries.

**Figure Supp2:** Gene copy numbers for 8 *sox*B phylotypes in “mix” (black), “rhizosphere” (dark grey) and “root” samples (light grey), measured in October at 5 cm depth. Values are represented as mean ± s.e.m. (n=3), with statistical differences determined by ANOVA and post-hoc Tukey tests (p=0.05) between sample types indicated by different letters for each site (lower case for Site 1, upper case for Site 2).

**Figure Supp3:** Transcript numbers for 8 *sox*B phylotypes in “mix” (black), “rhizosphere” (dark grey) and “root” samples (light grey), measured in October at 5 cm depth. Values are represented as mean ± s.e.m. (n=3), with statistical differences determined by ANOVA and post-hoc Tukey tests (p=0.05) between sample types indicated by different letters for each site (lower case for Site 1, upper case for Site 2).

**Figure Supp4:** Gene copy numbers for 5 *rdsr*AB phylotypes in “mix” (black), “rhizosphere” (dark grey) and “root” samples (light grey), measured in October at 5 cm depth. Values are represented as mean ± s.e.m. (n=3), with statistical differences determined by ANOVA and post-hoc Tukey tests (p=0.05) between sample types indicated by different letters for each site (lower case for Site 1, upper case for Site 2).

**Figure Supp5:** Transcript numbers for 5 *rdsr*AB phylotypes in “mix” (black), “rhizosphere” (dark grey) and “root” samples (light grey), measured in October at 5 cm depth. Values are represented as mean ± s.e.m. (n=3), with statistical differences determined by ANOVA and post-hoc Tukey tests (p=0.05) between sample types indicated by different letters for each site (lower case for Site 1, upper case for Site 2).
